# Anticancer Activities of Surfactin and Potential Application of Nanotechnology Assisted Surfactin Delivery

**DOI:** 10.3389/fphar.2017.00761

**Published:** 2017-10-26

**Authors:** Yuan-Seng Wu, Siew-Ching Ngai, Bey-Hing Goh, Kok-Gan Chan, Learn-Han Lee, Lay-Hong Chuah

**Affiliations:** ^1^School of Pharmacy, Monash University Malaysia, Bandar Sunway, Malaysia; ^2^Faculty of Science, School of Biosciences, The University of Nottingham Malaysia Campus, Semenyih, Malaysia; ^3^Centre of Health Outcomes Research and Therapeutic Safety (Cohorts), School of Pharmaceutical Sciences, University of Phayao, Phayao, Thailand; ^4^Global Asia in the 21st Century Platform, Asian Centre for Evidence Synthesis in Population, Implementation and Clinical Outcomes, Health and Well-being Cluster, Monash University Malaysia, Bandar Sunway, Malaysia; ^5^Division of Genetics and Molecular Biology, Faculty of Science, Institute of Biological Sciences, University of Malaya, Kuala Lumpur, Malaysia; ^6^Vice Chancellor Office, Jiangsu University, Zhenjiang, China; ^7^Advanced Engineering Platform, Monash University Malaysia, Bandar Sunway, Malaysia

**Keywords:** surfactin, lipopeptide, biosurfactant, anticancer, nano-formulation

## Abstract

Surfactin, a cyclic lipopeptide biosurfactant produced by various strains of Bacillus genus, has been shown to induce cytotoxicity against many cancer types, such as Ehrlich ascites, breast and colon cancers, leukemia and hepatoma. Surfactin treatment can inhibit cancer progression by growth inhibition, cell cycle arrest, apoptosis, and metastasis arrest. Owing to the potent effect of surfactin on cancer cells, numerous studies have recently investigated the mechanisms that underlie its anticancer activity. The amphiphilic nature of surfactin allows its easy incorporation nano-formulations, such as polymeric nanoparticles, micelles, microemulsions, liposomes, to name a few. The use of nano-formulations offers the advantage of optimizing surfactin delivery for an improved anticancer therapy. This review focuses on the current knowledge of surfactin properties and biosynthesis; anticancer activity against different cancer models and the underlying mechanisms involved; as well as the potential application of nano-formulations for optimal surfactin delivery.

## Introduction

Cancer remains the second highest cause of death worldwide, which contributed to 8.8 million deaths in 2015 (WHO, 2017) and could potentially reach 17 million new cases by 2020 (Obtel et al., 2015). Chemotherapy remains one of the mainstream anticancer treatments. About 60% of anticancer drugs are of natural origins, such as plants, microorganisms, vertebrates, and invertebrates (Demain and Sanchez, 2009). A known limitation of chemotherapy is chemoresistance (Alfarouk et al., 2015). In addition, many studies have proven that most chemotherapeutic drugs are highly cytotoxic and target highly proliferative cells in a non-specific manner, resulting in only a trivial improvement in patient survival (Sak, 2012; Dy and Adjei, 2013). This then leads to poor prognosis of cancer patients. In this regard, the search and development of new anticancer agents that selectively target cancer cells and sensitize the chemoresistant cancer cells are desirable (Gudiña et al., 2016). Among various sources for anticancer drugs, microorganisms have attracted much attention due to their ease in production manipulation and the potential to produce diverse bioactive metabolites, such as antibiotic, enzyme inhibitors, and biosurfactants (Chiewpattanakul et al., 2010). In fact, many studies have shown that biosurfactants are among the microbial metabolites that show promising biological activities (Dey et al., 2015).

Biosurfactants are defined as surface-active structurally different organic compounds produced by prokaryotic and eukaryotic microorganisms. They have been highlighted in recent years to be used as anticancer agents regulating cancer progression processes (Gudiña et al., 2013). These compounds are generally localized on the microbial surface and made of amphiphilic molecule, comprising both hydrophobic and hydrophilic moieties (Banat et al., 2010). Increasing evidence have shown that biosurfactants are superior to synthetic surfactant, owing to their microbial origin, high biodegradability and low toxicity, which are proven by their application in various biomedical fields (Marchant and Banat, 2012b). On the basis of their chemical structure and mode of action, they are divided into low (including glycolipids and lipopeptides) and high (including polysaccharides, proteins, lipoproteins, and among others) molecular weight biosurfactants (Gudiña et al., 2013; Liu et al., 2015). Comparatively, low molecular weight biosurfactants have greater surface-active activities as they possess a simpler chemical structure, with surfactin (lipopeptide) being the most widely studied biosurfactant (Fracchia et al., 2015).

Surfactin, a highly potent biosurfactant, is produced by *Bacillus subtilis* strains (Carrillo et al., 2003). Since its discovery as a macrolide lipopeptide, many lipopeptide antibiotics have been discovered (Kakinuma et al., 1969). It is still receiving much attention for its numerous physiological and biochemical activities due to its multifaceted interactions with biological systems (Seydlova and Svobodova, 2008). Surfactin has been reported to exhibit pharmacological actions, such as antibacterial and antifungal (Das et al., 2008), antimycoplasma (Boettcher et al., 2010), antiviral (Seydlova et al., 2011; Sachdev and Cameotra, 2013; Singla et al., 2014), anti-inflammatory (Byeon et al., 2008; Zhang et al., 2015), and thrombolytic (Kikuchi and Hasumi, 2002; Singla et al., 2014) properties. More recently, it has been shown to exert cytotoxic effects against many cancer types, such as Ehrlich ascites, breast and colon cancers, leukemia and hepatoma (Gudiña et al., 2016). Given its amphiphilic nature, the anticancer activity is attributed to the hydrophobic interaction of fatty acid moiety and the acyl chain of the membrane-bound phospholipids (Liu X. et al., 2010), while its peptide moiety interacts with cancer cells by binding to the polar heads of the membrane lipids (Liu X. et al., 2010). Furthermore, it was suggested that the delivery of surfactin in a liposome may reduce its toxicity while enhancing its therapeutic effect (Bouffioux et al., 2007), indicating the potential application of nano-formulation for surfactin.

This review presents a detailed understanding of surfactin in terms of structure and physicochemical properties; isolation and production; biosynthesis mechanisms and their regulations; and surfactin-membrane interactions. Furthermore, it highlights the recent findings on anticancer activity and the underlying mechanisms of surfactin against different cancer types. The potential application of nano-formulations for surfactin delivery is also discussed.

## Definition of biosurfactant

Biosurfactants are amphiphilic compounds produced by both prokaryotic and eukaryotic microorganisms (Sen, 2010). They contain both hydrophobic and hydrophilic moieties, in which the hydrophobic group usually consists of hydrocarbons while the hydrophilic group can be non-ionic, negatively or positively charged, or amphoteric (Sineriz et al., 2001). Due to its amphiphilic property, they are surface-active compounds, commonly referred to as surfactants. Surfactants can modify the conditions prevailing at interfaces, in which they tend to align naturally at the interface between two phases that differ in polarity and hydrogen bonding, such as oil/water or air/water (Rosenberg and Ron, 1999). In practice, biosurfactants decrease the free energy at the interface by replacing the bulk molecules with higher energy, thus reducing the interfacial tension. Surface tension is a measure of the amount of free energy required to bring a molecule from the bulk phase to the surface, which determines the effectiveness of a biosurfactant (Mulligan, 2005). Furthermore, the alteration of surface tension is dependent on the critical micelle concentration (CMC). CMC is the minimum concentration of a surfactant required to initiate micelle formation, and additional surfactant added to the system will spontaneously form micelles (Seydlova and Svobodova, 2008). It is also a representation of the maximum concentration of biosurfactant monomers in water that is influenced by pH, temperature, and ionic strength (Seydlova and Svobodova, 2008). Taken together, biosurfactants with low CMC have greater surface tension reducing effect as less biosurfactant is required.

One unique example of such versatile biosurfactant is surfactin, produced by several strains of Bacillus genus during the stationary phase where the nutrients and oxygen in the culture media are limited (Seydlova et al., 2011). Accumulating evidence have demonstrated that surfactin acts as a very powerful biosurfactant due to its amphiphilic nature containing a polar amino acid head and a hydrocarbon ring (Heerklotz and Seelig, 2001; Carrillo et al., 2003). CMC of surfactin in 200 mM NaHCO_3_ at pH 8.7 and 10 mM Tris, 10 mM NaCl at pH 8.5 are 9.4 and 7.5 μM, respectively (Carrillo et al., 2003). On the other hand, the effectiveness of surfactin as a potent surface-active agent was elucidated. It was found to reduce the surface tension of water from 72 to 27 mN m^−1^ at concentration as low as 0.005% (10 μM) in distilled water (Arima et al., 1968), which is significantly lower than CMC in water (23 mg/l) and two-folds lower than any other detergents (Heerklotz and Seelig, 2001). Moreover, it was also shown to have greater surface activity in comparison to its synthetic counterpart, sodium lauryl sulfate (Sen, 2010). Additionally, many investigations have shown that the biodegradability of most anionic lipopeptides, such as surfactin, is susceptible to chemical reaction and degradation under physiological conditions due to the presence of aspartic acid (Asp)-glycine segments in the peptide moiety (Geiger and Clarke, 1987; Seydlova and Svobodova, 2008).

The role of surfactin in bacterial physiology has been studied extensively by using a defective surfactin production machinery, in which many biological functions of *B. subtilis* strains were impaired (Kinsinger et al., 2005; Seydlova and Svobodova, 2008). One example was its loss of ability to form swarming colonies on the solid media (Banat et al., 2010). Some other possible roles of surfactin include the ability to increase the surface area of hydrophobic water-insoluble growth substrates (for example nutrients), thus leading to higher bioavailability of nutrients and also influences the ability of microorganisms' attachment and detachment from the growth substrates (Rosenberg and Ron, 1999). Besides its role as a potent biosurfactant, surfactin also exhibits some other biological activities, including antibacterial, antifungal, antimycoplasma, antiviral, fibrin clot inhibition, erythrocytes lysis (hemolytic activity), and more recently anticancer activity (Meena et al., 2016). Presumably, these activities are a direct consequence of the interaction of surfactin with its target membrane and the alteration of the phospholipid bilayers (Carrillo et al., 2003).

## Structure and physicochemical properties of surfactin

**Figure 1:**
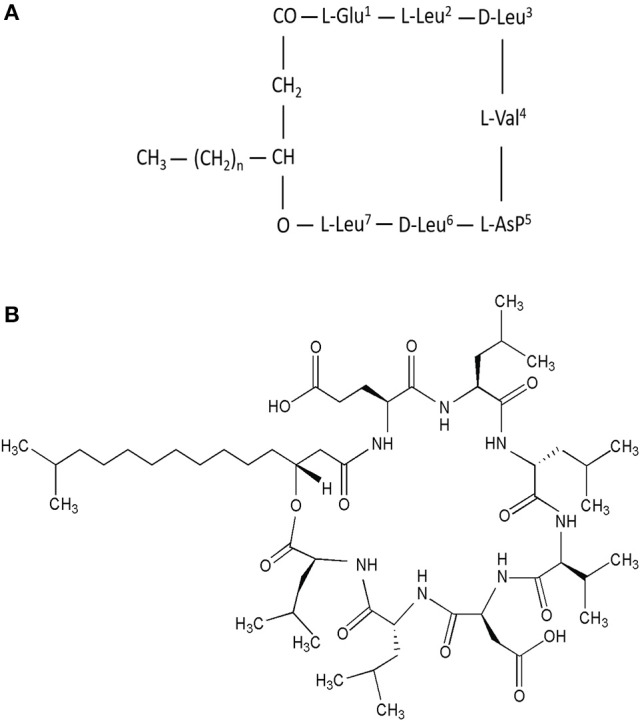
**(A)** Primary structure of surfactin, *n* = 9–11 (indicating the number of CH2 group in the peptide chain). **(B)** Chemical structure of surfactin. Seven amino acids are arranged in the cyclic ring connected with a fatty acid (β-hydroxy) of the chain lengths 12–16 carbon atoms to form a cyclic lactone ring.

Surfactin is an amphipathic cyclic lipopeptide with the molecular weight of 1,036 Da. It is constituted by a heptapeptide (ELLVDLL) with the chiral sequence LLDLLDL interlinked with a fatty acid chain of (β-hydroxy) of C12-C16 carbon chain which forms a close cyclic lactone ring structure (Figure 1). Hydrophobic amino acids of surfactin molecule are located at positions 2, 3, 4, 6, and 7 while hydrophilic glutamyl and aspartyl residues are located at position 1 and 5, giving the molecule two negative charges. Surfactin isoforms coexist in the cells as a mixture of seven peptide variants with a different aliphatic chain length. The molecular assembly of surfactin molecule in the aqueous solution or at the interface of air and water exploits the β-sheet structure, forming a horse-saddle conformation, which is believed to contribute to its wide biological activities.

**Figure 2:**
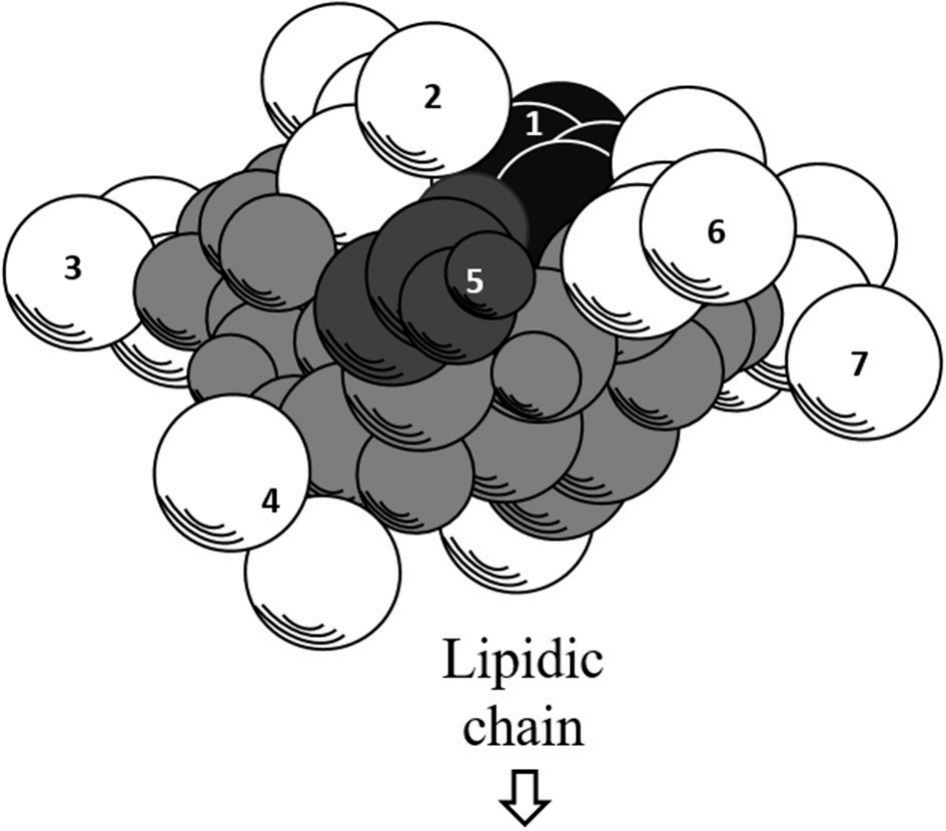
Three-dimensional structure of surfactin. The backbone is represented by the gray atoms. Seven amino residues (1–7) are presented. Hydrophobic residues (2, 3, 4, 6, and 7) are represented by white atoms, where the lipidic chain attaches. The black and dark gray atoms represent acidic residues 1 and 5, respectively.

Further exploration of its three-dimensional structure (Figure 2) using high-resolution 1H NMR incorporated with molecular imaging techniques has identified a minor polar domain at one side of the molecule formed by residues 2 and 6 that face each other near acidic Glu-1 and Asp-5 side chains. On the opposite side, residue 4 slightly faces the hydrophobic domain containing residues 3 and 7, making its amphiphilic nature and strong surface activity. At concentration lower than CMC, the lipidic chain is freely extended in the solution but it is still able to strongly involve in hydrophobic interactions, such as lipid micelles or oligomers at air/water interface. Thus far, there is no report of intrinsic water solubility and lipophilicity (Log P) of surfactin. However, surfactin has a hydrophilic-lipophilic balance (HLB) of 10–12, indicating it can freely dissolve in a mixture of water and oil phases (Gudiña et al., 2013). Furthermore, it is also soluble in aqueous solution optimally at pH 8–8.5 (alkaline waster) and organic solvents, such as ethanol, methanol, butanol, chloroform, and dichloromethane (Abdel-Mawgoud et al., 2008).

## Surfactin-membrane interaction

The biological activities of surfactin, including anticancer activities are determined by the interactions with biological membranes. In fact, the profile of membrane lipids in normal and cancer cells differs. For example, phosphatidylcholine, which plays a key role in stabilizing the bilayer structure, was found to be the major phospholipid component in cancer cells but not in normal cells (Preetha et al., 2005; Hilvo et al., 2011). In general, surfactin destabilizes the membrane and disrupts its integrity (Bernheimer and Avigad, 1970) via several hypothetical mechanisms, such as insertion into lipid bilayers, modification of membrane permeabilization via channel formation or diffusion of mono- and divalent ions across the membrane barrier and membrane solubilization by a detergent-like mechanism (Bouffioux et al., 2007).

Surfactin penetrates spontaneously into lipid membranes by means of hydrophobic interactions, which causes rearrangement of hydrocarbon order and membrane thickness (Maget-Dana and Ptak, 1995). In the lipid bilayer, surfactin displays a conformational change of peptide cycle which further mediates the interaction process (Maget-Dana and Ptak, 1995). Many studies have shown that the sensitivity of lipid membrane to surfactin is mainly determined by the lipid composition, polar head group region, length of fatty acid acyl chain, and lipid organization (e.g., physical state) (Kell et al., 2007; Buchoux et al., 2008; Deleu et al., 2013). In fact, the anticancer activity of surfactin is related to its hydrophobic nature (Gudiña et al., 2016). To penetrate the outer sheet of lipid bilayer, fatty acid moiety of surfactin strongly interacts with the acyl chain of the phospholipids whereas the peptide moiety interacts with the polar head group of the lipids in cancer cells. Furthermore, the extent of surfactin penetration is directly proportional to the chain length of peptide moiety, with C15 chain surfactin possessing greater anticancer activity than C13 and C14 chains (Liu X. et al., 2010).

In addition, surfactin concentration is critical for its perturbation effect on the membrane (Liu J. et al., 2010; Deleu et al., 2013). Surfactin could penetrate readily and miscibly with phospholipids at low concentrations, resulting in micelle formation. Ion-conducting pores in the membrane can be formed by domain segregation of surfactin at moderate concentrations. At high concentrations, surfactin has a prevailing detergent effect that can disrupt membrane easily (Grau et al., 1999). Studies have also shown that high concentrations of surfactin permits its insertion into phospholipid bilayers, inhibiting phase separation and consequently, solubilizes the phospholipid (Kell et al., 2007; Deleu et al., 2013). Moreover, the local surfactin-to-lipid (Rb) in the lipid bilayer necessary to induce leakage is Rb ~0.05 (Heerklotz and Seelig, 2007).

Surfactin can also drive mono- and divalent cations across an organic barrier, with divalent cations being transported more efficiently (Thimon et al., 1992). Na^+^ and K^+^ ions partially neutralize the two acidic residues, Glu-1 and Asp-5, at the air/water interface (Maget-Dana and Ptak, 1992), while Ca^2+^ ions induce complete neutralization. The cation chelation leads to inhibition of the cyclic AMP phosphodiesterase activity (Hosono and Suzuki, 1983; Seydlova and Svobodova, 2008). Apart from this, Ca^2+^ ions facilitate a deeper surfactin penetration into the membrane by neutralizing the charges of both surfactin and lipids in the subphase (Maget-Dana and Ptak, 1995; Grau et al., 1999), with the help of Glu-1 and Asp-5 forming a well-suited “claw” to stabilize the 1:1 surfactin-Ca2^+^ complex by means of an intramolecular bridge (Maget-Dana and Ptak, 1992).

## Biosynthesis of surfactin

### Microbial system of surfactin production

*B. subtilis* are rod-shaped Gram-positive bacteria found naturally in soils and plants. This bacteria species can grow under mesophilic temperatures of 25–37°C and possess a high survivability under stress, such as a nutrient deficient environment. Surfactin was firstly isolated from *B. subtilis* strains as white needle crystals in the search of fibrin clot inhibitor, with *B. subtilis* IFO 3039, IAM 1069, IAM 1213, IAM 1259, and IAM 1260 producing a fair amount (50–100 μg/ml) of this compound in nutrient broth in a 24-h bacterial culture. Besides, the ability to synthesize surfactin is also widely found in *B. pumilus, B. mojavensis, B. licheniformis, B. circulans, B. natto*, and *B. amyloliquefaciens* strains (Chen et al., 2015). Intriguingly, amongst all these species, *B. amyloliqufaciens* reportedly synthesized the highest amount of surfactin (4,525 μg/ml) in a study that collected 20-field Bacillus sp. isolates native in Taiwan (Hsieh et al., 2004).

Numerous studies have reported the successful production of surfactin (Wei et al., 2003, 2004; Yeh et al., 2005). Factors that could affect the optimal production of surfactin include cultivation conditions, media formulations and fermentation strategies (Chen et al., 2015). In contrast, efficient purification and isolation are critical for the recovery and concentration of surfactin (Chen et al., 2015).

### Biochemistry and mechanism

The biosynthesis of surfactin occurs via a non-ribosomal mechanism catalyzed by surfactin synthetase, an example of multienzymatic thiotemplates (Kluge et al., 1988; Shaligram and Singhal, 2010). In theory, the biosynthesis is based on the multiple- and subsequent-carrier concept, in which multiple 4'-phosphopantetheinyl cofactors (ppan) acts as an acceptor of the growing peptide chain and as a donor of the peptides to the next thiolester-linked amino acid in the template sequence. ATP-dependent adenylation is then used to activate all of the seven amino acids constituting the heptapeptide in surfactin before tethering to the distinct enzymatic modules via a carboxythioester bond (Nakano and Zuber, 1990; Vater et al., 1997).

**Figure 3:**
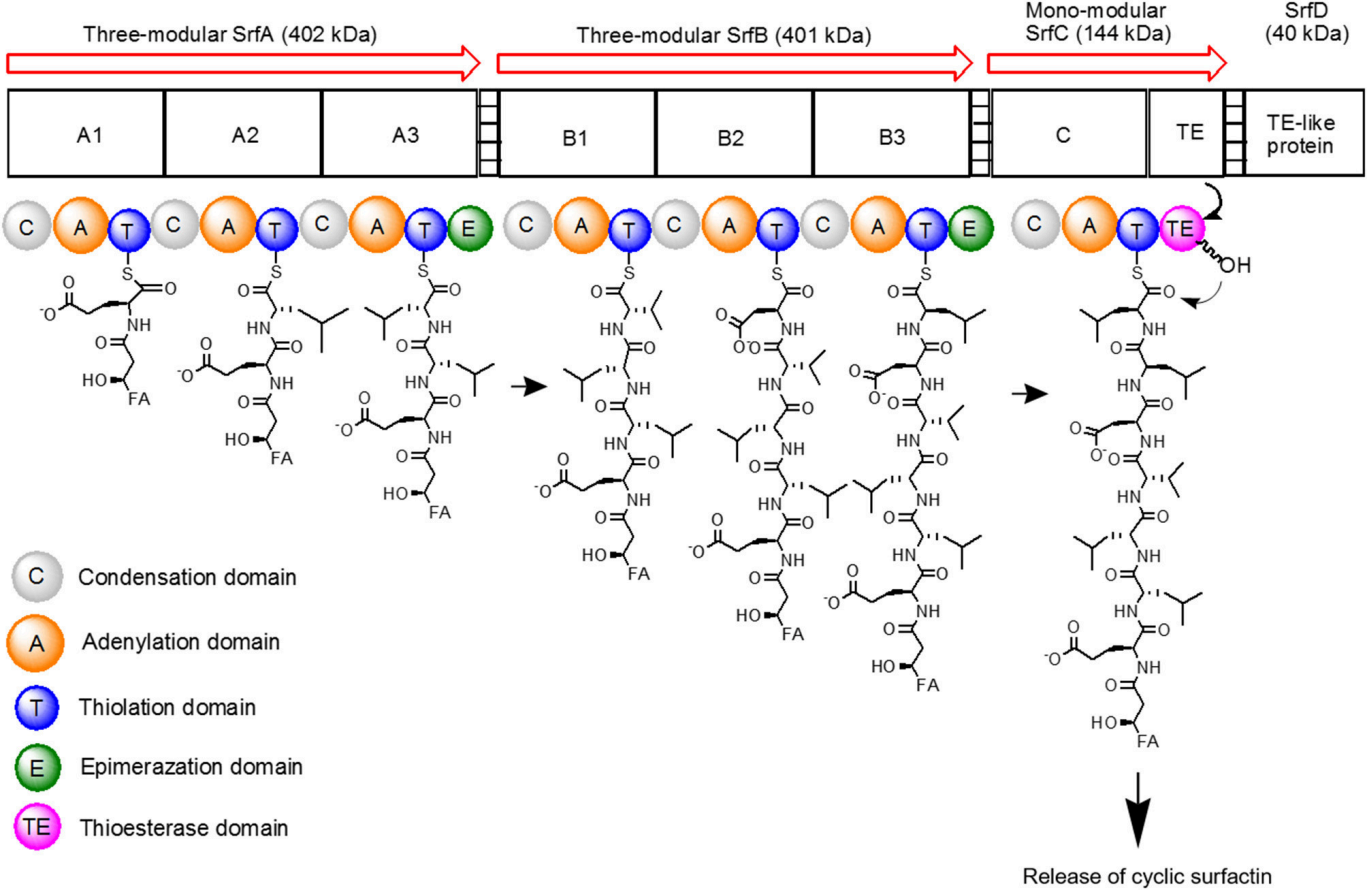
Schematic diagram of surfactin synthetase complex for biosynthesis of cyclic surfactin. Surfactin synthetase complex is composed of three-modular SrfA, three-modular SrfB, mono-modular SrfC and SrfD subunits, which is used to synthesize seven amino acids of surfactin. The peptide chain is elongated from left to right until the linear product is cyclized by TE domain.

The surfactin synthetase complex comprises of four subunits, such as SrfA (E1A, 402 kDa), SrfB (E1B, 401 kDa), SrfC (E2, 144 kDa), and SrfD (E3, 40 kDa) (Steller et al., 2004; Chen et al., 2015). SrfA, SrfB, and SrfC form seven modules that contain 24 catalytic domains, with each module functioning to incorporate one dedicated substrate to the growing heptapeptide chain (Peypoux et al., 1999). On the other hand, SrfD is responsible for surfactin initiation reaction (Steller et al., 2004). The surfactin synthetase and the biosynthesis steps of surfactin are as shown in Figure 3.

To initiate surfactin biosynthesis, SrfD protein firstly mediates the transfer of the β-hydroxy fatty acid substrate to the N-terminal L-Glu-activating module of surfactin synthetase to induce β-hydroxyacyl-glutamate formation (Steller et al., 2004). The substrate is then recognized and activated by the adenylation domain (A domain with about 550 amino acids) (Conti et al., 1997), resulting in aminoacyladenylate formation via Mg^2+^-dependent hydrolysis and pyrophosphate release (Dieckmann et al., 1995). The initiation products are then elongated by SrfA and SrfB catalyzation via thioester bond cleavages and simultaneous transpeptidation reactions (Peypoux et al., 1999). The aminoacyladenylate intermediates then bound to the free thiol group of the ppan cofactor before tethering to thiolation domain (T domain or peptidyl carrier protein with ~80 amino acids) (Weber et al., 2000). Following, the intermediates bound to the ppan cofactor can undergo subsequent catalytic reactions by other domains. In regard to regulation mechanisms, acyltransferase enzyme eliminates incorrect charging of the ppan cofactor from the reaction centers by using its external thioesterase activity (SrfTE-II) during the initiation process (Schwarzer et al., 2002).

Lastly, SrfC subunit catalyzes the condensation of the last amino acid residue via peptide bond formation between two adjacent substrates using condensation domain (C domain with ~450 amino acids) (Peypoux et al., 1999; Kraas et al., 2010). Furthermore, SrfC also catalyzes the release of lipoheptapeptidyl intermediate from the surfactin synthetase complex that is stimulated by thioesterase domain (TE with ~280 amino acids) embedded in SrfC. The resulting peptide is either released as a linear acid by hydrolysis or as a cyclic peptide by an intramolecular reaction with a nucleophile (Peypoux et al., 1999).

In addition to A, T, C, and TE domains, there are other optional domains with a distinctive role. For example, epimerization of domain E (with ~450 amino acids) catalyzes the racemization of T domain-bound amino acid, whereby T domain-bound L-Leu requires two E domains catalyzation from modules 3 and 6. Then, the adjacent C domain transfers D-amino acid onto the growing peptide chain. In combination with D- and L-amino acids, this gives rise to a peptide with a unique conformation that interacts specifically with the cell membranes (Linne and Marahiel, 2000; Seydlova and Svobodova, 2008). Compared to surfactin biosynthesis, its excretion process and the involved mechanism are still elusive. Nonetheless, an assumption has been suggested that surfactin can be excreted from its producers by passively diffusing across the cytoplasmic membrane as no active transporter has been identified (Tsuge et al., 2001).

### Regulation of biosynthetic genes in surfactin biosynthesis

Genetic analysis plays a prerequisite role in controlling or regulating the surfactin biosynthesis (Sen, 2010). In fact, the assembly of surfactin synthetase complex is reflected in the chromosomal organization of its genes (Seydlova and Svobodova, 2008). Surfactin synthesis is controlled by two genetic loci, namely srf and sfp (Sen, 2010). The surfactin synthetase complex is coded by the inducible operon, srfA locus (25 kb) with four modular open reading frame (ORF), such as ORF1 (srfAA), ORF2 (srfAB), ORF3 (srfAC), and ORF4 (srfAD) that encode the four surfactin subunits (Hamoen et al., 2003). The srfB locus has a similar function with comA locus that is responsible for genetic competence in *B. subtilis* (Nakano and Zuber, 1989; Nakano et al., 1992). The srfA encodes some enzymes that are involved in catalyzing the surfactin synthesis (Nakano et al., 1991). For example, srfAA encodes an enzyme that contains the amino acid-activating domain for Glu, Leu, and D-Leu while the amino-acid domains for Val, Asp, and L-Leu are found in an enzyme encoded by srfAB. In addition, srfAC is responsible for encoding an enzyme that contains L-Leu amino acid-activating domain. Lastly, srfAD encodes an enzyme that resembles the primary structure of thioesterases family, with more sequence homologies with type II thioesterases (Fuma et al., 1993; Peypoux et al., 1999; Kraas et al., 2010; Satpute et al., 2010).

Surfactin biosynthesis is also influenced by the expression of sfp gene that mapped at 4 kb downstream of srfA locus. It encodes an enzyme with 224 amino acids that belongs to the superfamily of 4'-phosphopantetheinases (Nakano et al., 1992). The sfp enzyme has two functions, in which it can be used as the primers for non-ribosomal peptide synthesis, as well as for producing cofactors containing holoform from inactive surfactin synthetase apoform (Lambalot et al., 1996).

In addition to its role in surfactin biosynthesis, srfA gene expression also correlates with the development of genetic competence of *B. subtilis* in response to exogenous DNA uptake in the stationary phase cultures of glucose-grown cells (D'Souza et al., 1993). An example is that a nutritional stress during the late exponential phase could stimulate the global regulatory mechanisms, ComP-ComA and Spo0A-AbrB, thereby inducing the expression of srfA (Hamoen et al., 2003). Other than that, comS gene that is located within or outside the srfA gene frame is also involved in *B. subtilis* competence development (Hamoen et al., 2003).

## Anticancer properties of surfactin

**Figure 4:**
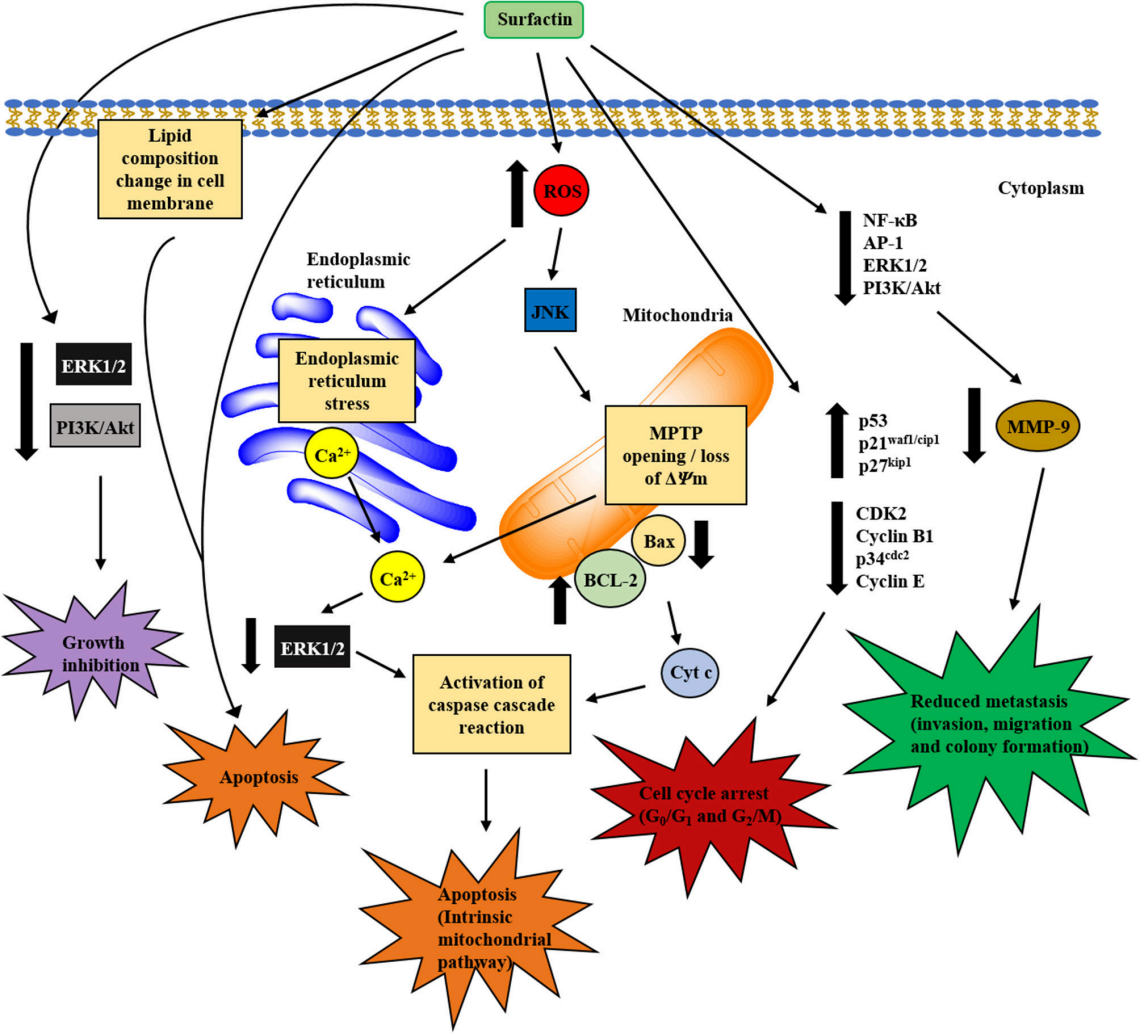
Proposed mechanisms involved in *in vitro* anticancer activity of surfactin. The anticancer activity of surfactin is associated with growth inhibition, cell cycle arrest, cell death (apoptosis), and metastasis inhibition. Surfactin treatment can inhibit cancer cell viability by inactivating the cell survival signaling pathways. Besides, surfactin regulates cell cycle-regulatory proteins, which are pivotal for cell cycle phase transition to block the proliferation of cancer cells. The apoptotic effect (intrinsic mitochondrial/caspase pathway) of surfactin is mediated by two different pathways that are triggered by high intracellular ROS formation, namely ERS/[Ca^2+^]_i_/ERK1/2 and JNK/ΔΨm /[Ca^2+^]_i_/Bax-to-Bcl-2 ratio/cyt c pathways. Surfactin-induced apoptosis is also associated with the changes in phospholipids composition that leads to a significant decrease in unsaturated degree of cellular fatty acids. Apart from these, surfactin also inhibits the invasion, migration and colony formation of cancer cells in the virtue of MMP-9 expression change that involves the inactivation of NF-κB, AP-1, PI3K/Akt, and ERK1/2 signaling pathways.

The structural and amphiphilic properties of surfactin have widely contributed to its biological activities, including anticancer activity (Wang et al., 2009). In fact, the anticancer activity of surfactin on a wide range of cancer cell lines has been studied recently, including antiproliferative and apoptotic effect, with its inhibitory action on cancer metastasis (Park et al., 2013). Surfactin-mediated programmed cell death is mainly caused by inducing apoptosis, which plays an important role in the regulation of tissue development and homeostasis, and presents a promising strategy for cancer treatment (Sergeev, 2005). Surfactin-mediated cytotoxicity on different cancer models (Ehrlich ascites, breast and colon cancers, leukemia, hepatic, cervical, etc.) (Table 1) and the molecular mechanisms involved in inducing apoptosis are presented in Figure 4.

**Table 1:** Anticancer activity of surfactin or surfactin-like biosurfactants against cancer cells.

**Cancer type**	**Cell line**	**Surfactin Origin**	**Isolation and Purification Method**	**Activity**	**Assay**	**Treatment Dose (h)**	**Normal cell line (IC_50_)**	**References**
Ehrlich ascites	Ehrlich ascites	*B. natto* KMD 1126	Acidic precipitation, ethyl acetate (AcOEt) extract, sephadex G-25, sephadex LH-20, and crystallization	Cytolytic activity	Cylinder plate	–	–	Kameda and Kanatomo, 1968
		*B. natto* KMD 2311	Acidic precipitation, AcOEt extract, sephadex G-25, sephadex LH-20, and crystallization	Cytolytic activity	Cylinder plate	–	–	Kameda et al., 1974
Breast	MCF-7	*B. subtilis* CSY 191	Acidic precipitation, centrifugation, methanol (MeOH) extract, thin-layer chromatography (TLC), and reversed-phase high performance liquid chromatography (RP-HPLC) system	Growth inhibition	MTT	IC_50_ = 9.65 μM (24 h)	–	Lee et al., 2012
		*B. subtilis natto* TK-1	Acidic precipitation, MeOH extract, TLC, and C_18_ RP-HPLC system	Growth inhibition	MTT	IC_50_ = 86.2 μM (24 h), 27.3 μM (48 h), 14.8 μM (72 h)	HEK 293T (No IC_50_)	Cao et al., 2009
				Cell cycle arrest	Flow cytometry and western blotting (p53, p21, p34^cdc2^, and cyclin B1)	27.3 μM	–	
				Apoptosis induction	Acridine orange/ethidium bromide staining, TUNEL assay, analysis of [Ca^2+^]_i_		–	
		*B. subtilis natto* TK-1	Acidic precipitation, MeOH extract, TLC, and C_18_ RP-HPLC system	Apoptosis induction	DCFH-DA (ROS measurement), analysis of ΔΨm, caspase-6 activity, MTT (after NAC treatment in surfactin-treated cells), and western blotting (ERK1/2, p38, and JNK)	30 μM	–	Cao et al., 2010
		*B. subtilis natto* TK-1	Acidic precipitation, MeOH extract, TLC, and C_18_ RP-HPLC system	Growth inhibition	MTT	IC_50_ = 29 μM (48 h)	–	Cao et al., 2011
				Apoptosis induction	DCFH-DA (ROS measurement), analysis of [Ca^2+^]_i_, analysis of MPTP, and ΔΨm, caspase-9 activity, and western blot (cyt c)	29 μM		
		*B. subtilis*	Commercially available	No growth inhibition or cytotoxicity	MT	10 μM	–	Park et al., 2013
				Inhibition of invasion, migration, and colony formation	Wound healing, Matrigel invasion, gelatin zymography, RT-PCR western blotting (MMP-2, MMP-9, c-Jun, c-Fos, p65, and IκB-α), transient transfection, immunofluorescence, chromatin immunoprecipitation (p65 and AP-1), and dual luciferase			
	T47D	*B. subtilis* 573	Acidic precipitation, centrifugation, demineralized water dissolution, and freeze-drying	Growth inhibition	MTS	IC_50_ = 93 μM (48 h)	MC-3 T3-E1 (93 μM at 72 h)	Duarte et al., 2014
				Cell cycle arrest	Flow cytometry		–	
	MDA-MB-231	*B. subtilis*	Commercially available	No growth inhibition or cytotoxicity	MT	10 μM	–	Park et al., 2013
				Inhibition of invasion, migration, and colony formation	Wound healing, Matrigel invasion, gelatin zymography, RT-PCR western blotting (MMP-2, MMP-9, c-Jun, c-Fos, p65, and IκB-α), transient transfection, immunofluorescence, chromatin immunoprecipitation (p65 and AP-1), and dual luciferase			
		*B. subtilis* 573	Acidic precipitation, centrifugation, demineralized water dissolution, and freeze-drying	Growth inhibition	MTS	IC_50_ = 93 μM (72 h)	MC-3 T3-E1 (93 μM at 72 h)	Duarte et al., 2014
				Cell cycle arrest	Flow cytometry	48 μM	–	
	Bcap-37	*B. subtilis* Hs0121	Acidic precipitation, centrifugation, lyophilization, MeOH extract and C18 RP-HPLC	Growth inhibition	MTT	IC_50_ = 29 ± 2.4 μM (24 h)	HaCaT (97 μM at 24 h)	Liu X. et al., 2010
				Apoptosis induction (fatty acid composition change)	Surface tension measurement, flow cytometry (propidium iodine staining), nuclei staining, and GC/MS (fatty acid analysis)	12–96 μM	-	
Colon	HCT15	*B. circulans* DMS-2	Acidic precipitation, alkaline water dissolution, lyophilization, and MeOH extract, HPLC system	Growth inhibition	MTT	IC_50_ = 77 μM (24 h)	NIH/3T3 (482 μM at 24 h)	Sivapathasekaran et al., 2010
	HT29			Growth inhibition	MTT	IC_50_ = 116 μM (24 h)	NIH/3T3 (482 μM at 24 h)	Sivapathasekaran et al., 2010
	LoVo	*B. subtilis*	Commercially available	Growth inhibition	MTT	IC_50_ = 26 μM (48 h)	–	Kim et al., 2007
				Cell cycle arrest	Flow cytometry (Annexin V/PI staining) and RT-PCR (p53, p21^waf/cip1^, CDK2, and cyclin E)	30 μM		
				Apoptosis induction	RT-PCR (Fas R, Fas L, Bax) and western blotting (PARP, cleaved-caspase 3, ERK, p38, JNK, p85, and Akt)			
Leukemia	K562	*B. subtilis natto* T-2	Acidic precipitation, MeOH extract, charcoal treatment, Pharmadex LH 20, and C_18_ HPLC system	Growth inhibition	MTT	IC_50_ = 10–20 μM (24, 36 and 48 h)	–	Wang et al., 2007
				Cell cycle arrest	Flow cytometry and western blotting (cyclin D1, p21^waf/cip1^, and p27)	7.7 μM		
				Apoptosis induction	Nuclei staining, caspase-3 activity, and western blotting (caspase-3 and PARP)			
		*B. subtilis natto* T-2	Acidic precipitation, MeOH extract, charcoal treatment, Pharmadex LH 20, and C_18_ HPLC system	Apoptosis induction	TUNEL staining, lactate dehydrogenase measurement, analysis of [Ca^2+^]_i_ and western blotting (ERK, p38, JNK, Bax, Bcl-2, cyt c, and caspase-3)	15.4 μM	–	Wang et al., 2009
Hepatocellular	BEL7402	*B. subtilis* HSO121	Acidic precipitation, centrifugation, lyophilization, MeOH extract, and C_18_ RP-HPLC system	Growth inhibition	MTT	IC_50_ = 35 ± 12 μM (24 h)	HaCaT (97 μM at 24 h)	Liu X. et al., 2010
	HepG2	*B. natto* TK-1	Acidic precipitation, MeOH extract, TLC, and C_18_ RP-HPLC system	Apoptosis induction	DCFH-DA (ROS measurement) and analysis of [Ca^2+^]_i_,	41 μM	–	Wang et al., 2013
Cervical	HeLa	*B. subtilis* HSO121	Acidic precipitation, centrifugation, lyophilization, MeOH extract, and C_18_ RP-HPLC system	Growth inhibition	MTT	IC_50_ = 37 ± 4.5 μM (24 h)	HaCaT (97 μM at 24 h)	Liu X. et al., 2010
		*B. subtilis*	Commercially available	Growth inhibition	MTT	IC_50_ = 86.9 μM (16 h), 73.1 μM (24 h), 50.2 μM (48 h)	HaCaT (97 μM at 24 h)	Nozhat et al., 2012
Oral epidermoid	KB-3-1	*B. subtilis* HSO121	Acidic precipitation, centrifugation, lyophilization, MeOH extract, and C_18_ RP-HPLC system	Growth inhibition	MTT	IC_50_ = 57 ± 2.6 μM (24 h)	HaCaT (97 μM at 24 h)	Liu X. et al., 2010
Pancreatic	SW-1990	*B. subtilis* HSO121	Acidic precipitation, centrifugation, lyophilization, MeOH extract, and C_18_ RP-HPLC system	Growth inhibition	MTT	IC_50_ = 58 ± 1.6 μM (24 h)	HaCaT (97 μM at 24 h)	Liu X. et al., 2010
Rat melanoma	B16	*B. subtilis* HSO121	Acidic precipitation, centrifugation, lyophilization, MeOH extract, and C_18_ RP-HPLC system	Growth inhibition	MTT	IC_50_ = 20 ± 1.5 μM (24 h)	HaCaT (97 μM at 24 h)	Liu X. et al., 2010

### Ehrlich ascites carcinoma

Kameda and his collaborators carried out two studies to investigate the anticancer activity of surfactin against Ehrlich ascites carcinoma, which was originally established as an ascites tumor in mice. Surfactin isolated from a strain of *B. natto* (tentatively called KMD 1126) showed *in vivo* anticancer activity on Ehrlich ascites tumor (Kameda and Kanatomo, 1968). In 1974, Kameda et al. further revealed that *B. natto* KMD 2311 had stronger cytolytic activity against Ehrlich ascites carcinoma as tested using cylinder plate method among 113 strains of *B. natto* isolated from straws in Japan (Kameda et al., 1974).

### Breast cancer

Among all cancer types tested, the anticancer activity of surfactin on breast cancer cell lines has been most widely studied. *B. subtilis* CSY 191-derived surfactin was found to inhibit the growth of human MCF-7 breast cancer cells in a dose-dependent manner, with IC_50_ of 9.65 μM at 24 h. Higher surfactin production, which was obtained from co-fermentation of cheonggukjang and strain CSY 191, further enhanced the level of anticancer activity from 2.6- to 5.1-fold compared to surfactin produced by strain CSY 191 alone (Lee et al., 2012). Another strain of *B. subtilis* 573 also significantly reduced the cell viability of T47D and MDA-MB-231 in a dose- and time-dependent manner, with 50% reduction (at 193 μM) for T47D and MDA-MD-231 at 48 and 72 h, respectively. Furthermore, inhibition of cell proliferation had a 10% increase in cell cycle arrest at G0/G1 phase after surfactin treatment (193 μM). It was also reported that 50% of normal MC-3T3-E1 breast cell growth were inhibited at the same dose (Duarte et al., 2014).

Furthermore, surfactin-like lipopeptides purified from *B. subtilis* Hs0121 also showed potent cytotoxicity on human Bcap-37 breast cancer cells. The results showed that C15 surfactin-like lipopeptide exerted the highest inhibition on the cell viability, with IC_50_ of 29 ± 2.4 μM after 24 h exposure. The apoptotic effect induced was associated with a significant decrease in the unsaturated degree of the cellular fatty acids in Bcap-37 cells due to a reduction in the amount of fatty acids, thereby enhancing membrane fluidization (Liu X. et al., 2010).

Surfactin isolated from *B. subtilis natto* TK-1 strains was demonstrated to inhibit the proliferation of MCF-7 cells in a dose- and time-dependent manner, with IC_50_ at 24, 48, and 72 h being 86.2, 27.3, and 14.8 μM, respectively. The cytotoxicity was tumor-selective as surfactin treatment (100 μM) only reduced 20% viability of normal human HEK 283T embryonic cells. Surfactin also increased intracellular calcium concentration [Ca^2+^]_i_ and induced apoptosis by arresting cells at G2/M phase up to 47% at 48 h (Cao et al., 2009). Indeed, surfactin also led to the accumulation of tumor suppressor p53 and cyclin kinase inhibitor p21^waf1/cip1^, and inhibited the activity of G2-specific kinase, cyclin B1/p^34cdc2^ (Cao et al., 2009), all of which are important for cell cycle phase transition. Cao et al. (2010) further demonstrated that surfactin-induced apoptosis occurred via a reactive oxygen species/c-Jun N-terminal kinase (ROS/JNK)-mediated mitochondrial/caspase pathway. Surfactin treatment (30 μM) stimulated high ROS generation, leading to mitochondrial permeability and membrane potential (ΔΨm) loss via JNK phosphorylation. These activities further led to cytochrome c (cyt c) release and caspase-cascade reaction (Cao et al., 2010).

*B. subtilis natto* TK-1-purified surfactin can also induce a similar underlying mechanism of ROS/Ca^2+^-mediated mitochondrial/caspase pathway to stimulate apoptosis. MCF-7 cells treated with surfactin (29 μM) caused 50% viability inhibition while 80% inhibition was observed at 68 μM. Intriguingly, only 15% inhibition of normal human L-02 hepatic cell viability was observed at the highest dose tested (97 μM), indicating surfactin's non-toxicity to normal cells. High ROS generation was observed in surfactin-induced apoptosis, which is reversible by antioxidant N-acetylcysteine. The results further indicated that surfactin treatment initially induced ROS formation, leading to mitochondrial permeability transition pore (MPTP) opening and ΔΨm collapse. The resultant increase in [Ca^2+^]_i_ caused by the changes in mitochondrial permeability was inhibited by 1,2-bis (2-aminophenoxy) ethane-N,N,N',N'-tetraacetic acid (BAPTA-AM, a calcium chelator). These activities led to the release of cyt c and activated caspase-9, later inducing apoptosis (Cao et al., 2011).

Owing to the efficacy of surfactin-induced growth inhibition, further investigation into breast cancer metastasis using *in vitro* model was reported recently. Surfactin inhibited 12-O-tetradecanoylphorbol-13-acetate (TPA)-induced migration, invasion and colony formation by downregulating matrix metalloprotenaise-9 (MMP-9) expression at 10 μM, a dose lower than the suboptimal dose that had no cytotoxic effect on MCF-7 and MDA-MB-231 cells. Surfactin attenuated TPA-induced activation and nuclear localization of nuclear factor-kappa B (NF-κB) and activating protein-1 (AP-1), and strongly repressed phosphatidylinositol 3-kinase (PI3K)/Akt and extracellular signal-regulated kinase (ERK) signaling pathways. In fact, PI3K/Akt and ERK signaling pathways are involved in multiple cellular processes, such as cell survival, proliferation, apoptosis, and cell cycle (Gudiña et al., 2013). Altogether, surfactin-mediated inhibition of cell invasion and MMP-9 expression involves the regulation of NF-κB, AP-1, PI3K/Akt, and ERK pathways (Park et al., 2013).

### Colon cancer

In the literature, two investigations into surfactin-mediated anticancer activity on colon cancer have been reported. A study showed that surfactin was able to inhibit the proliferation of LoVo colon cancer cells significantly in a dose- and time-dependent manner, with IC_50_ of 26 μM at 48 h. The antiproliferative action induced by surfactin was mediated by apoptotic effect as shown by DNA fragmentation, morphological change, altered levels of apoptosis-, and cell cycle-regulatory proteins. For example, upregulation was observed for Fas receptor (Fas R), Fas ligand (Fas L), cleaved poly (ADP-ribose) polymerase (PARP), cleaved caspase-3, p53 and p21^waf1/cip1^ while CDK2 and cyclin E were downregulated in a dose- and time-dependent manner. In addition, surfactin treatment led to 10% of G0/G1 phase cell cycle arrest and induction of 40% apoptotic cells. Besides, the phosphorylation levels of PI3K/Akt signaling were also suppressed upon surfactin treatment. These data suggest that surfactin-mediated growth inhibition was associated with apoptosis induction and cell cycle arrest via the inhibition of PI3K/Akt cell survival signaling (Kim et al., 2007).

Another study showed that purified surfactin-like lipopeptides isolated from a marine *B. circulans* DMS-2 induced moderate cytotoxicity against HCT-15 and HT-29 colon cancer cells, with respective IC_50_ of 77 and 116 μM after 24 h treatment. At a higher dose of 290 μM, 90% inhibition of cell viability was observed. This study also demonstrated the anticancer activity of surfactin is tumor-selective—a high concentration of surfactin (482 μM) was required to inhibit 50% of normal mouse NIH/3T3 embryo fibroblast cell viability. It was reported that the crude biosurfactant displayed very low cytotoxicity against cancer cell lines, which is possibly due to the relatively low amount of surfactin-containing lipopeptides inside (Sivapathasekaran et al., 2010). It was further reported that IC_50_ induced by purified lipopeptides is comparable to the IC_50_ of surfactin (30 μM) (Kim et al., 2007).

### Leukemia

Crude cyclic lipopeptides (CLPs) purified from *B. subtilis natto* T-2, with surfactin as a main constituent displayed cytotoxicity against human K562 leukemia cells at varying concentrations (2–62 μM) for 24, 36, and 48 h. CLPs inhibited the growth of K562 cells in a dose- and time-dependent manner, with the IC_50_ value between 10 and 20 μM for 24–48 h treatment period. In contrast, the viability of normal HLF primary lung fibroblast was not affected by CLPs when the concentration is lower than 30 μM. The antiproliferative activity of surfactin was associated with cell cycle arrest and apoptosis induction. Surfactin treatment resulted in a notable accumulation of cells in the G1 phase (36.5–57.6%) and increased the number of apoptotic cells. Apoptotic activities induced by surfactin include morphological change and enhanced the apoptosis-related proteins, such as caspase-3, cleaved PARP, p21^waf1/cip1^, and p27^kip1^ while remarkably reduced the expression of cyclin D1, which plays a role to regulate G1 phase progression (Wang et al., 2007).

Wang et al. (2009) further investigated the underlying mechanism of apoptosis contributing to the anticancer activity of surfactin. They revealed that surfactin induced apoptosis via [Ca^2+^]_i_/ERK-mediated mitochondrial/caspase pathway. Sustained increase in [Ca^2+^]_i_ was observed after treatment of surfactin (15 μM) for 3–9 h. Increased [Ca^2+^]_i_ was associated with cell apoptosis and ERK phosphorylation. CLPs-induced apoptosis in K562 cells was inhibited by PD98059 (an ERK inhibitor), but displayed no effect with p38 and JNK inhibitors, indicating ERK signaling as a critical role in apoptosis induction. It was also shown that apoptosis rate was partially decreased after reducing the [Ca^2+^]_i_ with 1,2-Bis(2-aminophenoxy)ethane-N,N,N′,N′-tetraacetic acid tetrakis(acetoxymethyl ester) (BAPTA-AM) pretreatment. Collectively, this study showed that the phosphorylation of ERK signaling caused by increased [Ca^2+^]_i_ activated Bax, cyt c, and caspase 3, leading to apoptosis (Wang et al., 2009).

### Hepatocellular carcinoma

Surfactin has also been shown to exhibit cytotoxic effect on hepatocellular carcinoma. Liu X. et al. (2010) demonstrated that the purified surfactin-like lipopeptides isolated from *B. subtilis* HSO121 significantly inhibited the cell viability of human Bel-7402 hepatoma cells, with IC_50_ of 35 ± 12 μM compared to normal human HaCaT keratinocyte cells (IC_50_ of 97 μM) at 24 h, indicating the specificity of anticancer action toward Bel-7402 cells. Furthermore, Wang et al. (2013) recently proposed the signaling network underlying the apoptosis of HepG2 hepatoma cells induced by surfactin, resembling the mechanisms identified in a breast cancer model. Sustained ROS generation and [Ca^2+^]_i_ accumulation upon surfactin treatment (40 μM) were responsible for apoptosis induction. The release of Ca^2+^ ions from inositol 1,4,5-trisphosphate and ryanodine receptors channels contributed to increased [Ca^2+^]_i_, which in turn stimulated endoplasmic reticulum stress (ERS). Meanwhile, ERS was also induced by surfactin-produced ROS. Collectively, activated ERS reversibly increased [Ca^2+^]_i_ and reduced the phosphorylation levels of ERK signaling pathway (Wang et al., 2013). Therefore, these findings suggest that surfactin induces apoptosis in HepG2 cells via ROS/ERS/Ca^2+^-mediated ERK pathway.

### Cervical cancer

The anticancer activity of surfactin on cervical cancer was mainly tested on HeLa cell line. The studies were limited to examining the inhibitory effect of surfactin on cell viability with the underlying mechanism being investigated. Liu X. et al. (2010) showed that purified surfactin-like lipopeptides dose-dependently inhibited the viability of HeLa cancer cells, causing 50% reduction at 37 ± 4.5 μM after 24 h exposure. With increasing attention to the application of diverse biocompatible nanoparticles in cancer treatment, surfactin C15 nanopeptide has been shown to induce cytotoxicity in HeLa cells (Liu X. et al., 2010). With this approach, the cell viability was reduced with increasing nanopeptide concentrations and exposure times. The IC_50_ of surfactin causing 50% cell viability inhibition for 16, 24, and 48 h was 86.9, 73.1, and 50.2 μM, respectively. Further investigations exploring the molecular mechanisms underlying the anticancer activity of surfactin on HeLa cells as well as testing the potential cytotoxic effect of surfactin on other cervical cancer cell lines are desirable.

### Other cancer types

The inhibitory effect of surfactin or surfactin-like biosurfactants against the growth of other cancer types, such as human oral epidermoid carcinoma, pancreatic, and rat melanoma cancer has also been investigated. However, the assessment of the anticancer potential for this compound is only limited to examining the metabolic activity of cancer cells using 3-(4,5-dimethylthiazol-2-yl)-2,5-diphenyl tetrazolium bromide (MTT) assay. For example, surfactin-like lipopeptide was found to cause cytotoxicity against oral epidermoid (KB-3-1), pancreatic (SW-1990), and rat melanoma (B16) cancer after 24 h exposure, with their respective IC_50_ was 57 ± 2.6, 58 ± 1.6, and 20 ± 1.6 μM (Liu X. et al., 2010).

Based on the studies on surfactin's anticancer activity against different cancer models, surfactin is mainly isolated and purified from several strains of *B. subtilis* and *B. natto*, and one strain of *B. circulans*. Among these Bacillus sp., *B. subtilis*-produced surfactin has the highest cytotoxic effect against cancer cells followed by *B. natto* and *B. circulans*, which may be due to their origin and variations in cultivation, isolation, and purification methods. Although some researchers have shown the potential anticancer promoting activity of surfactin, only few studies have further investigated the mechanisms underlying surfactin-induced apoptosis. Some of these studies use a single cancer cell line to examine the cytotoxicity of surfactin, without using normal cell lines as controls. It is inconclusive as to whether the anticancer effect is tumor-selective or only specific against the single cell line studied. In addition, a few studies using a single method to evaluate the inhibitory effect of surfactin on cancer cell proliferation, such as metabolic measurement assays, which are mainly used to measure the metabolic activity of viable cells could not truly represent the proliferation state of cancer cells after surfactin treatment. Furthermore, some promising results obtained in the *in vitro* study could not fully claim their potential anticancer activity, thus further validation by means of *in vivo* experiments are warranted.

## Toxicity of surfactin

There have been several studies on the anticancer effects of surfactin against cancer cell lines that indicate its selective cytotoxicity toward cancer cells. Nonetheless, surfactin purified from *B. subtilis* 573 was shown to induce cytotoxicity against human normal MCT-3T3-E1 fibroblast cell line at the same concentrations and exposure times that inhibited the viability of human T47D and MDA-MB-231 breast cancer cells (Duarte et al., 2014). Hwang et al. (2009) tested the acute toxicity of surfactin C by oral administration to rats with different doses. At high dose of 1,000 or 2,000 mg /kg, increased serum levels of Alanine transaminase (ALT), Aspartate transaminase (AST), and alkaline phosphatase (ALP) were observed, indicating necrosis of hepatocyte. At lower dose, surfactin C did not show any toxic effects during treatment period, and the no-observed-adverse-effect level (NOAEL) of surfactin C was determined to be 500 mg/kg (Hwang et al., 2009).

One of the major drawback of surfactin's use as an anticancer agent is its hemolytic activity above 0.05 g/l (Dehghan-Noude et al., 2005). To overcome this, linear forms of surfactin have been developed, and shown to be non-hemolytic while protecting erythrocytes against the detergent-like action of surfactin (Seydlova and Svobodova, 2008). No significant hemolysis was observed when linear surfactin was used up to 1,000 μM. Moreover, a protective effect against Triton X-100 induced hemolysis was found. The concentration at which this protective effect happens is directly correlated to the CMC of the linear surfactin, and inversely correlated to the acyl chain length of the product.

As the toxicity of surfactin may bring about serious side effects, cautious use of surfactin at safe doses is crucial. Although chemical modification of cyclic surfactin into linear structure has been shown to have reduced toxicity, the therapeutic effects of this linear compound is still unknown. In this regard, nano-formulations may be a more promising alternative for surfactin delivery for reduced toxicity and enhanced anticancer effects.

## Potential nano-delivery of surfactin

Delivery of surfactin nano-formulations for anticancer treatment has been largely unexplored to date. This section provides the insights of surfactin nano-formulations in the literature, and discusses the potential of such formulations to be extended to anticancer therapeutic delivery of surfactin. A summary of these nano-formulations can be found in Table 2.

**Table 2:** The functions and applications of surfactin in different nano-formulations.

**Nano-formulation**	**Type of nano-formulation**	**Surfactin function**	**Application of nano-formulation**	**References**
Surfactin-loaded polyvinyl alcohol (PVA) nanofiber	Polymeric nanofibers	As active	Wound dressing, anti biofilm	Ahire et al., 2017
Surfactin-functionalized poly(methylmethacrylate) (PMMA) nanoparticles	Polymeric nanoparticles	Enhance sorption activities	Adsorbent, antibacterial	Kundu et al., 2016
Poly(methyl methacrylate) (core)–biosurfactant (shell) nanoparticles	Polymeric nanoparticles	Emulsifier, pH responsive gate keeper, Control release of drugs	pH responsive and controlled release nanocarrier	Hazra et al., 2014b
Surfactin nanomicelles	Micelles	As building blocks for micelles, As active	Anticancer	Nozhat et al., 2012
Mixed surfactin-sodium dodecylbenzenesulphonate (SDOBS) micelles	Polymeric micelles	Surface active agent	–	Onaizi et al., 2012
Surfactin-containing self-microemulsifying system (SMEDDS)	SMEDDS	As active	–	Kural and Gürsoy, 2011
Cooking oil-encapsulated nanoemulsion	Nanoemulsion	Emulsifier	Antibacterial, antifungal	Joe et al., 2012
Cationic 1,2-dioleoyl-sn-glycero-3-ethylphosphocholine (EDOPC)-based surfactin liposome	Liposome	Enhance cellular uptake of siRNAs into cells	siRNAs delivery system to cancer cells	Shim et al., 2009
CaSO_4_ nanocrystals	Inorganic nanocrystals	Prevent particle aggregation and precipitation of CaSO_4_	Filler of nanocomposite materials	Hazra et al., 2014a
Anionic surfactin-mediated silver nanoparticles	Inorganic nanoparticles	Stabilizer	–	Reddy et al., 2009b
Anionic surfactin-mediated gold nanoparticles	Inorganic nanoparticles	Stabilizer	–	Reddy et al., 2009a
Cadmium sulfide nanoparticles	Inorganic nanoparticles	Stabilizer, capping agent	–	Singh et al., 2011
Surfactin-stabilized biogenic silver nanocubes	Inorganic nanoparticles	Stabilizer	Antipseudomonal, anti-endotoxin	Krishnan et al., 2017

### Surfactin and nano-formulations

The failure of conventional anticancer treatment are largely due to non-specific distribution of drugs in the body, leading to reduced efficient dose to the cancer cells, causing suboptimal results, and excessive toxicity to normal cells (Khodabandehloo et al., 2016). In recent years, nano-formulations using nano-sized carriers (10–400 nm) have made considerable contributions in cancer therapy. This claim is based on the advantages of nano-formulations offering high drug loading capacity, improved bioavailability, prolonged circulation time, better cancer targeting, and the ease of manipulating drug release (Yu et al., 2010). To date, a variety of nanocarriers have been developed, including but not limited to, polymeric nanoparticles, micelles, dendrimers, liposomes, niosomes, solid lipid nanoparticles, gold nanoparticles, nanotubes, magnetic nanoparticles, protein nanoparticles, micro-, and nano-emulsions (Cho et al., 2008; Yuan et al., 2016). The efficacy of these nanocarriers in delivering drug compounds has been tested using both *in vitro* and *in vivo* models (Davis et al., 2008; Jagur-Grodzinski, 2009).

By virtue of the small size, nano-formulations are able to accumulate preferentially at the cancer site via the enhanced permeation and retention (EPR) effect. Due to the rapid growth of cancer cells, the blood vessels around the tumor are poorly formed, leading to its leaky nature with wider gaps, allowing nanoparticles to pass through. At the same time, the lymphatic system draining the tumor is impaired. This results in the lack of drainage of macromolecules out of the tumor (Greish, 2010). Together, both constitute the EPR effect. Nano-formulations of surfactin could make use of this EPR effect for enhanced delivery to the cancer cells. However, in order to achieve the EPR effect, prolonged circulation of nano-formulations in the body is desired. Upon entering the blood circulation, the nano-formulations would face the risk of opsonization and subsequent recognition by the reticulo-endothelial system (RES), leading to its clearance out of the body. To avoid this, the nano-formulations carrying surfactin can be surface-decorated with hydrophilic polymer, such as polyethylene glycol (PEG). The high affinity of PEG to water could attract water molecules to the nano-formulation, shielding the nano-formulations from being detected by the RES (Morachis et al., 2012). This results in prolonged circulation of the surfactin nano-formulations, increasing its accumulation at the tumor site via the EPR effect.

Surface functionalization of nano-carriers can also achieve target specificity in anticancer treatment. During cancer angiogenesis, various receptors are upregulated on the tumor cells for uptake of more nutrients (Zwicke et al., 2012). Overexpression of these receptors became an opportunity for cancer targeting. To effectively deliver surfactin to the tumor, the nano-formulations carrying surfactin can be surface modified with targeting ligands. Having high affinity to the overexpressed receptors, these targeting ligands would drive the surfactin-loaded nano-formulations toward the cancer cells, delivering a high amount of surfactin to the cancer cells instead of normal cells. The combined effect of EPR and ligand- receptor targeting would result in increased surfactin concentration in the tumors cells, possibly leading to an improved treatment efficacy.

Nano-carriers also offer the advantage of protecting the drug against premature burst release, resulting in reduced toxicity. Modification of the nano-formulations provides a controlled release of surfactin, such that the dose is released slowly over a period of time, ensuring small dose release at a time, to minimize the toxicity associated with surfactin. This can be achieved by formulating surfactin into polymeric nanoparticles. Careful control of the polymeric network density permits slow release of surfactin out of the nanoparticles over time. This is especially important to curb the hemolytic side effects of surfactin.

The biochemical, physicochemical, and self-assembling properties of biosurfactants have attracted much attention in the application of nanotechnology to treat various diseases (Rodrigues, 2015). Biosurfactants can be incorporated into nanocarriers, functioning either as a surfactant or stabilizer in the development of nanocarriers (Gudiña et al., 2013; Rodrigues, 2015). Besides, biosurfactants that possess various biological activities, including anticancer treatment, could be delivered to the target site using nanocarriers. Given the amphiphilic nature, surfactin could effortlessly achieve optimal incorporation into the nanocarriers. The following sections discuss the incorporation of surfactin in different nano-formulations, as well as the different roles that surfactin play in these formulations.

### Surfactin as an active in nano-formulations

#### Surfactin in polymeric nanoparticles and nanofibers

Polymeric nanoparticles (NP) are submicron-sized colloidal particles. Loading of drugs onto the NP can be achieved via adsorption on the NP surface; or encapsulated within the NP. The polymer network could protect the drug against degradation activities by enzymes found in the body (Bei et al., 2010). Drug release from the NP can take place by diffusion, hydrolysis of the polymeric network, enzymatic degradation of the polymer, or a combination of different mechanisms. Polymeric materials used in pharmaceutical applications are often biodegradable in nature, which include synthetic polymers such as poly(lactide) (PLA), poly(lactic-co-glycolide) (PLGA), poly(ε-caprolactone) (PCL); and natural polymers like chitosan, alginate, and albumin (Banik et al., 2016).

**Figure 5:**
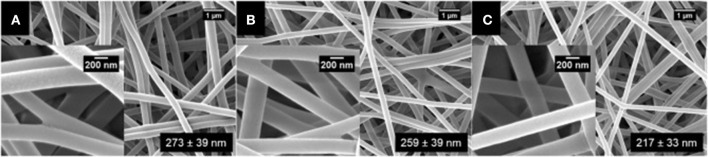
SEM images of **(A)** PVA (10%, *w*/*v*) loaded with **(B)** 0.5% (*w*/*v*), **(C)** 1.0% (*w*/*v*), and **(D)** 1.5% (*w*/*v*) surfactin (Adopted from Ahire et al., 2017). Reprinted with permission. Copyright (2017) Elsevier.

Surfactin-loaded polyvinyl alcohol (PVA) nanofiber was formulated for wound dressing as well as to coat prothestic devices to prevent biofilm formation and secondary infection. The nanofibers were produced by mixing surfactin and PVA solutions, followed by gravity electrospinning. Increasing levels of surfactin was found to decrease the diameter of the nanofiber, as shown by the scanning electron micrographs (Figure 5) (Ahire et al., 2017). Although this nanofiber did not exhibit antimicrobial activity, it significantly reduced the adhesion of *Listeria monocytogenes* EDGe (Ahire et al., 2017).

When synthesizing surfactin-functionalized poly(methylmethacrylate) (PMMA) nanoparticles for sorption properties, Kundu et al. (2016) found that these nanoparticles displayed significant antibacterial activity against *Escherichia coli* at 300 μg/ml of the nanoparticles, due to the presence of surfactin. Hence these nanoparticles can be used for both sorption properties as well as for antibacterial effects. Moreover, the nanoparticles exhibited low hemolytic activity (Kundu et al., 2016). This shows the possibility of reduced surfactin-associated toxicity when this biosurfactant is incorporated into a nanocarrier, allowing a safer delivery of surfactin to the patients.

#### Surfactin in polymeric micelles

Polymeric micelles are made of amphiphilic copolymer with a core-shell structure. At concentration above the CMC, the amphiphilic polymers self-assemble into the micellar structure, with the outer shell being hydrophilic and inner core hydrophobic. The hydrophobic core could encapsulate poorly water-soluble drugs while the hydrophilic shell could help to solubilize the drug. Micelles can appear in various forms, such as spherical, disc-like, and cylindrical structures depending on the concentration (Akter et al., 2013).

**Figure 6:**
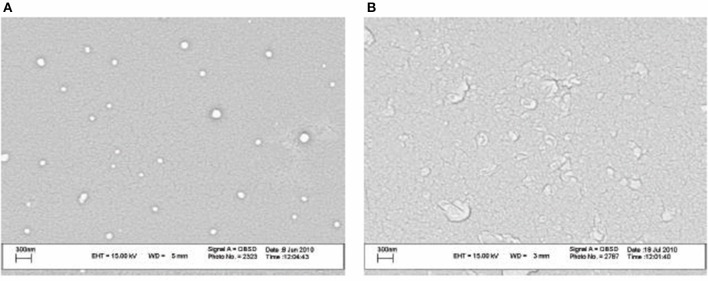
SEM images of surfactin nanomicelle in distilled water **(A)** and PBS **(B)** (pH 7.4) (Adopted from Nozhat et al., 2012). Reprinted from open access journal, Copyright (2012) Zahra Nozhat et al.

**Figure 7:**
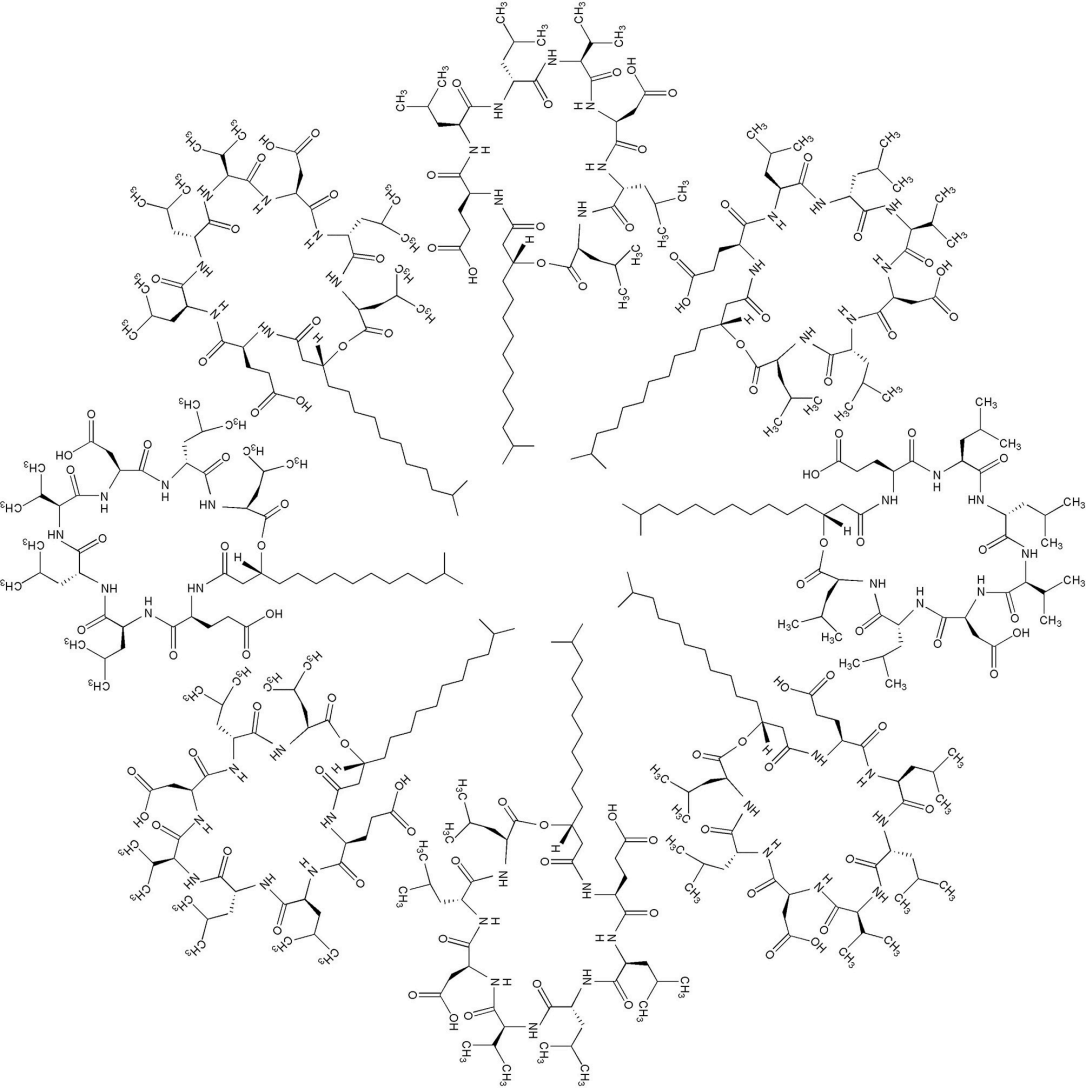
Proposed micelle formation using surfactin as building blocks.

Recently, Nozhat et al. (2012) formulated surfactin C15 nanopeptide into nanomicelles, and the scanning electron micrographs are shown in Figure 6. Apart from the ability to form nanomicelles, surfactin C15 nanopeptide was also shown to arrest the growth of HeLa cell line in a dose- and time-dependent manner. In fact, the ability of surfactin to form micelles came as no surprise due to its surfactant properties. Figure 7 shows a proposed micellar structure of surfactin. Other than forming micelles on its own, surfactin can also form mixed micelles with other surfactant, for example, the synthetic surfactant sodium dodecylbenzenesulphonate (SDOBS), as demonstrated by Onaizi (Onaizi et al., 2012). The mixed surfactin-synthetic surfactant may represent a greener and more sustainable formulation, along with reduced costs associated with the exclusive use of biosurfactants (Gudiña et al., 2013).

Pluronic block copolymers, which are composed of poly(ethylene oxide) (PEO) and poly(propylene oxide) (PPO) and arranged in PEO-PPO-PEO structure, have been widely used in drug delivery systems (Alakhova and Kabanov, 2014). Upon self-assembly, the core of the micelles will be composed of PPO blocks, which allow for the encapsulation of hydrophobic drugs. The outer shell is composed of hydrated PEO moieties, suitable for incorporation of hydrophilic drugs (Nambam and Philip, 2012). Formulation scientists can make use of this structure for surfactin delivery, as the compound could fit well in the micellar structure. Being amphiphilic in nature, the hydrophilic part of surfactin could sit in the hydrophilic shell of the micelles, with its hydrophobic part staying in the core.

#### Surfactin in microemulsions

Microemulsion is a colloidal-based drug delivery system, where two immiscible solvents are brought together, with a surfactant monolayer present at the interface. It is composed of an aqueous phase (water), a hydrocarbon (oil phase), a surfactant, and with or without a cosurfactant (Israelachvili, 1994; Akter et al., 2013). It has great ability to encapsulate or solubilize hydrophilic and hydrophobic drugs either as oil-in-water (O/W) or water-in-oil (W/O) microemulsions. There is potential of reducing toxic effects associated with the drug when it is present within the dispersed phase (Israelachvili, 1994; Damasceno et al., 2012). However, as microemulsions are highly thermodynamic-dependent, any changes in the composition could lead to phase separation and loss of emulsified drugs (Gudiña et al., 2013).

A stable self-microemulsifying drug delivery system (SMEDDS) formulation has been developed for the delivery of surfactin (Kural and Gürsoy, 2011). The optimum SMEDDS formulation was prepared by mixing PEG 3000, Gelucire 44/14, Labrasol and Vitamin E in the ratio of 1:1:8:0.5 (% *w*/*w*). Maintaining neutral pH is crucial for the surface activity of surfactin, as it tends to lose its surfactant properties at lower pH (Kim and Gates, 1997; Wei and Chu, 1998; Abdel-Mawgoud et al., 2008). The addition of surfactin into the formulation led to slight increase in the mean droplet size of the blank formulation (8.8 ± 0.03 nm) due to the rearrangement of surfactant (Kural and Gürsoy, 2011). Furthermore, the average zeta potential of surfactin-containing formulations was −2.72 ± 1.06 mV which could occur in the presence of anionic Glu/Asp residues in the peptide chain of surfactin (Kural and Gürsoy, 2011). The melting endotherm of surfactin was not observed in the thermogram of the surfactin-SMEDDS formulation, indicating the presence of strong interaction of surfactin with the excipients at the O/W interface (Kural and Gürsoy, 2011).

More recently, Joe et al. (2012) developed a surfactin-based nanoemulsion using cooking oils, such as sunflower, castor, coconut, groundnut, and sesame oils. The oil phase of the O/W nanoemulsion consists of 14% selected cooking oils, 3% ethanol, and 3% surfactin. The mean droplet size of different surfactin based cooking oil emulsions range from 72.52 to 875.22 nm, with the smallest size achieved with sunflower oil formulation (Joe et al., 2012). Further investigations showed that surfactin-based sunflower oil nanoemulsion demonstrate antibacterial activity against *Salmonella typhi, Listeria monocytogenes*, and *Staphylococcus aureus* (Joe et al., 2012). It also showed potent antifungal activity against *Rhizopus nigricans, Aspergillus niger*, and Penicillium sp. (Joe et al., 2012). This finding could lead to the potential use of this formulation as preservatives in food products.

#### Surfactin in liposomes

Liposomes are vesicles with a hydrophobic shell and a hydrophilic core, built as a spherical phospholipid bilayer. This amphiphilic structure enables the loading of both lipophilic and hydrophilic drugs into the liposome. The phospholipid bilayer in liposomes is composed of natural phospholipids, which are biologically inactive with minimal toxicity, and therefore have good biocompatibility (Sercombe et al., 2015). Liposomes are often characterized by its ease of preparation, straightforward drug encapsulation, non-immunogenicity as well as high drug solubility (Glaser et al., 2017). Liposomes with anionic membrane lipids, such as cardiolipin and phosphatidylserine, can prevent direct active drug efflux by P-glycoprotein, thus enhancing cellular absorption compared to free drugs (Kapse-Mistry et al., 2014).

There have been several studies on the interactions of surfactin with various lipid models, differing in chain length and polar head group, with an aim to understand the behavior of surfactin in the lipid matrix. Using dimyristoyl phospholipids, surfactin was found to have a strong interaction with dimyristoylphosphatidylcholine (DMPC) compared to L-α-dimyristoylphosphatidylethanolamine (DMPE) and L-α-dimyristoylphosphatidic acid (DMPA) monolayers due to electrostatic repulsion (Maget-Dana and Ptak, 1995). Surfactin penetration across the lipids is greater when the acyl chain length of the phospholipids decreases (Maget-Dana and Ptak, 1995; Liu X. et al., 2010). Furthermore, the miscibility between surfactin and phospholipids is higher for shorter acyl chains and greater polar head group of phospholipids. Surfactin also has a destabilizing effect on dipalmitoylphosphatidylcholine (DPPC); but it can stabilize dipalmitoylphosphatidylethanolamine (DPPE) and dipalmitoylphospharidylserine (DPPS) molecular interactions (Bouffioux et al., 2007). These findings indirectly contribute to design of efficient surfactin in liposomes delivery systems by manipulating the balance between the phospholipids composition in a liposome formulation.

To date, there is no reported development of surfactin-loaded liposome delivery system in the literature. Nonetheless, other lipopeptides such as marine somocystinamide A (ScA) has been successfully encapsulated into liposomes for anticancer treatment. ScA was able to completely partition into phospholipids of 100 nm liposome (nanosome) and alter the membrane structure, which was composed of cholesterol, 1,2-dioleoyl-*sn*-glycero-3-phosphoethanolamine, 1,2-dioleoyl-*sn*-glycero-3-phosphocholine (DSPC), scA, 1,2-dioleoyl-*sn*-glycero-3-phosphoethanolamine-*N*-[methoxy(polyethyleneglycol)-2000] ammonium salt at 1:1:1:0.16:0.16 molar ratio (Wrasidlo et al. (2008). Interestingly, ScA-loaded liposome induced cytotoxicity against a panel of cancer cell lines in a manner comparable to free ScA. ScA altered the lipid compartment by inducing ceramide formation and accumulation, which was associated with apoptotic caspase 8 induction (Wrasidlo et al., 2008).

The proven interaction between surfactin and phospholipids, along with the successful formulation of lipopeptide ScA into liposomes, provide a strong foundation for future development of surfactin-loaded liposomes. With a better understanding of formulation approaches and the characteristics of surfactin-loaded liposomes, this formulation would have great potential for anticancer treatment.

### Surfactin as a component of nano-carrier

Surfactants have traditionally been widely used in many drug delivery applications for their effects as wetting agents, solubilizing agents, surface tension reducer, emulsifier; as well as use in dispersion and micellization (Lawrence, 1994). Their applications are not limited to conventional dosage forms, but also extend to nano-formulations, such as in nano-micelles, nano-emulsions, niosomes, to name a few. In the quest for greener, sustainable and renewable resources, biosurfactants emerged as a decent alternative to replace synthetic surfactants (Marchant and Banat, 2012a). Surfactin, being derived from bacteria and having all desirable properties similar to other surfactants, present itself as the ideal choice.

Incorporation of surfactin into a cationic 1,2-dioleoyl-sn-glycero-3-ethylphosphocholine (EDOPC)-based liposome was shown to enhance the cellular delivery of small interfering RNAs (siRNA) to cancer cells. The cationic EDOPC-based liposomes were formulated using 3, 6, and 14 mol % surfactin in the lipid membrane of liposomes. The size of the lipoplexes was not more than 200 nm and the Zeta potential values were significantly reduced following complexation with siRNAs (Shim et al., 2009). The 14% surfactin-containing liposomes did not cause significant reduction in the viability of HeLa cells (84.0 ± 8.1%) compared to cells treated with surfactin-free liposomes (82.0 ± 0.5%) (Shim et al., 2009), proving that surfactin is safe at this concentration. The presence of surfactin in the liposomes was found to increase the extent of cellular delivery of siRNA in the Hela cell line in a concentration dependent manner. These findings indicated that cationic surfactin liposomes could be used as a biocompatible delivery system of nucleic acid based medicines, such as siRNA.

Some biosurfactants have been found to have membrane destabilizing effects at concentrations below their CMC. Their structure and nature allows them to interact with the lipid bilayer and cholesterol that sits in the membrane. Hence, some biosurfactants are used as penetration enhancers to transdermal delivery. Nicoli et al. (2010) investigated the potential use of surfactin to enhance skin delivery of aciclovir. Even though surfactin increased the concentration of aciclovir in the epidermis by 2-fold, enhanced transport of aciclovir across the skin was not seen (Nicoli et al., 2010). This shows that surfactin, or other lipopeptide biosurfactants, might not be suitable as penetration enhancers for transdermal delivery, despite having the ability to interact with the membrane.

The use of surfactin was also reported in the synthesis of calcium phosphate nanoparticles using reverse microemulsion. As a surface-active agent, surfactin plays a role in the formulation of emulsion to lower the interfacial tension, resulting in nano-sized droplets. Using different water/surfactin ratios, nanoparticles of diverse structures were produced, with the average particle size in the range of 16–200 nm (Maity et al., 2011). In surfactant-template/ultrasound-assisted nanocrystals synthesis, surfactin was used to control the morphology and aspect ratio of nano-calcium sulfate (CaSO_4_) by adjusting the mass ratio of surfactin/H_2_O and rhamnolipid/surfactin. With increasing surfactin concentration, the crystal morphology of nano-CaSO_4_ was found to undergo gradual changes from submicrometer-sized long rod to hexagonal plate, and then to plate-like appearance. Surfactin was shown to inhibit calcium sulfate precipitation, stabilizing the intermediate solution phases during production of the nanocrystals, resulting in nanocrystals of different morphology (Hazra et al., 2014a). The same group of researchers also synthesized PS(core)-biosurfactant (shell) nanoparticles as biocompatible and biodegradable drug delivery vehicle. Incorporation of biosurfactant improved the process of classical emulsion polymerization. The unique surfactin coated poly(methyl methacrylate) (nPMMA) have successfully resulted in a pH-responsive nanocarrier, along with a tailored drug release profile (Hazra et al., 2014b).

In general, HLB, critical packing parameter (CPP) and Winsor R ratio could be used to design a successful microemulsion (Gudiña et al., 2013). HLB affects the stability of the emulsion and the relative contribution of hydrophilic and lipophilic groups of a surfactant. Surfactants with low HLB (3–6) value indicate the tendency to form W/O microemulsions while high HLB (8–18) tends to form O/W microemulsions. For surfactants with high HLB value (>20), a cosurfactant is usually required to reduce their effective HLB (Gudiña et al., 2013). The CPP parameter is used to measure the ability of a surfactant to form aggregates with respect to its geometries (Gudiña et al., 2013). Winson R indicates the ratio of total energy (per unit area of the interface) for a surfactant to interact in O and W phases and is also dependent on environmental factors (Gudiña et al., 2013).

The ability of surfactin to be formulated into microemulsions can be determined by its experimental HLB and CPP values (Shen et al., 2011; Gudiña et al., 2013). Based on the HLB (10–12) and CPP (0.1435) values, surfactin could form O/W microemulsions when the alkaline condition is maintained. Only a small amount of surfactin is required to formulate a microemulsion for drug delivery as it has high surface activity (Gudiña et al., 2013).

### Surfactin as a stabilizer in the nano-formulations

In addition to acting as a vital component or enhancer during nanoparticles development, surfactin has also shown to function as a good stabilizing and capping agent in developing metal nanoparticles due to the surface-active properties. Reddy and team used different proton concentrations to cause the conformational change of surfactin that is used to stabilize gold and silver nanoparticles (Reddy et al., 2010). The synthesized nanoparticles were found to be stable for 2 months (Reddy et al., 2009a,b). Besides gold and silver nanoparticles, surfactin produced by *B. amyloliquifaciens* KSU-109 was also used as a stabilizer in cadmium sulfide nanoparticles (Cds-NPs). The Cds-NPs was synthesized by mixing 0.005% surfactin with 1 mM Cds in 1:1 ratio (*v*/*v*) and 10 mM sodium sulfide at pH 7.2. The Cds-NPs was found to be stable for 4 months (Singh et al., 2011). More recently, surfactin-producing *Brevibacillus brevis* KN8(2) was used to stabilize a nanocrystalline silver nanoparticles (AgNPs) (Krishnan et al., 2017). Surfactin-stabilized AgNPs induced a minimum inhibitory concentration of 10 μg/ml against *Pseudomonas aeruginosa*, which is a causative agent of diabetic foot infection. Additionally, the AgNPs ameliorated the *P. aeruginosa*-infected wounds of diabetic mice (Krishnan et al., 2017), suggesting that surfactin-stabilized AgNPs confers the treatment for gram negative bacteria-infected diabetic wounds.

### Surfactin nano-formulations for anticancer treatment

The great potential of surfactin for anticancer treatment not only comes from its proven cytotoxicity to cancer cells, but also due its multiple roles when incorporated in nano-formulations. The amphiphilic structure and surface-active properties of surfactin make it an ideal candidate to be incorporated into nano-formulations. Polymeric micelles, liposomes, and niosomes are particularly suitable, as surfactin can easily position itself within the hydrophobic/hydrophilic core-shell structure of such nano-formulations. Micro- and nano-emulsions are great choices too, where surfactin can be dispersed well in the formulation. Moreover, the use of surfactants is common as stabilizer and emulsifier in emulsions.

Apart from functioning as the active compound used for therapeutic purposes, surfactin can be incorporated to improve the formulation. For example, in a nanoemulsion, surfactin can be added to act as an emulsifier and an active for its anticancer activities. With this combination, the use of surfactin in the nano-formulation could then generate double actions for better delivery and enhanced efficacy of cancer treatment. In effect, it could even simplify the formulation process as less materials are needed to produce the nano-formulations, when surfactin can be the carrier, and the load at the same time.

Drug formulation scientists could also utilize the unique features of surfactin to incorporate it as one of the components of the nanocarrier, such as the use of surfactin as building blocks in the formulation of micelles, liposomes and niosomes. This can be exemplified by the surfactin nanomicelles mentioned in section Surfactin in Polymeric Micelles, where surfactin self assembles into micelles for its own delivery. In this regard, pure surfactin could be used without the need of other materials, ensuring the maximum dose of surfactin to be delivered to the cancer cells.

There has been increasing interests in combination therapy of multiple drugs incorporated in a single nano-formulation, giving rise to higher therapeutic effect. For example, a mesoporous silica nanoparticle was used to encapsulate topotecan and quercetin against triple negative breast cancer cells (MDA-MB-231) and multidrug resistance breast cancer cells (MCF-7). The treatment significantly enhanced the drug uptake efficiency by cancer cells while thwarted the uptake by normal cells (Murugan et al., 2016). This is also an area to explore for surfactin nano-formulations, whereby a combination of surfactin and another chemotherapeutic drug can be loaded into the nano-formulation, with surfactin acting as an adjuvant for anticancer treatment.

Surfactin nano-formulation is an area of great interest and significant potential for formulation scientists, yet remains largely undiscovered. There is ample space for research and development in utilizing surfactin nano-formulation as an improved nanomedicine strategy to combat cancer.

## Conclusion

In summary, numerous studies have shown surfactin's anticancer promoting activity against a few cancer types, many focusing on breast cancer. Surfactin-induced anticancer activities involve growth inhibition, cell cycle arrest, apoptosis, and metastasis arrest (invasion, migration, and colony formation). Growth inhibition caused by surfactin is mediated by reducing the PI3K/Akt and ERK cell survival signaling pathways. Besides, surfactin blocks the proliferation of cancer cells by regulating cell-cycle regulatory proteins such as tumor suppressor p53 and its downstream effector p21waf1/cip1, CDK2, cyclin B1, cyclin E, p34cdc2, and p27kip1. Surfactin inhibits cancer metastasis by downregulating MMP-9 expression that is mediated by NF-κB, AP-1, PI3K/Akt, and ERK1/2 inactivation. The mechanisms underlying surfactin-induced apoptosis are mainly associated with ROS formation, which could either induce ERS or ΔΨm change followed by increasing [Ca^2+^]_i_. Increased [Ca^2+^]_i_ stimulates the release of cyt c and activates the caspase-cascade reaction to induce apoptosis. It is notable that surfactin can also induce hemolytic activity and may selectively exert cytotoxicity to normal cells, all of which limits its application as an anticancer agent.

To improve the anticancer effect of surfactin, a promising strategy would be the use of nano-formulations due to their ability to achieve the drug selectivity to cancer cells with minimal toxicity to normal cells. Given its amphiphilic nature, surfactin presents itself as a good candidate for incorporation into various nano-formulations, such as polymeric NPs, micelles, microemulsions, liposomes, to name a few. Numerous surfactin nano-formulations have been reported in the literature. Surfactin nanomicelles (surfactin C15 nanopeptide) have been formulated and shown to induce cytotoxic effect on HeLa cancer cells. A new surfactin-SMEDDS formulation has been developed, though its anticancer activity requires further confirmation. To date, no liposome-borne surfactin formulation has been developed, even though surfactin could interact with different lipid models, implying its great potential to be incorporated into liposome. There have also been various surfactin nano-formulations reported designed for other purposes, where drug formulation scientists could pick these up and extend them into surfactin anticancer applications. In fact, this presents a great opportunity for further advances in surfactin nano-formulations. The potential improved anticancer activity of surfactin nano-formulations thus offers much room for establishment.

## Author contributions

The literature review and manuscript writing were performed by YW, LC, and SN, while BG, KC, and LL provided vital guidance and insight for the writing. The research topic was conceptualized by LC and BG.

### Conflict of interest statement

The authors declare that the research was conducted in the absence of any commercial or financial relationships that could be construed as a potential conflict of interest.

## References

[B1] Abdel-Mawgoud A. M. Aboulwafa M. M. Hassouna N. A. (2008). Characterization of surfactin produced by *Bacillus subtilis* isolate BS5. Appl. Biochem. Biotechnol. 150, 289–303. 10.1007/s12010-008-8153-z 18437297

[B2] Ahire J. J. Robertson D. D. van Reenen A. J. Dicks L. M. T. (2017). Surfactin-loaded polyvinyl alcohol (PVA) nanofibers alters adhesion of *Listeria monocytogenes* to polystyrene. Mater. Sci. Eng. C Mater. Biol. Appl. 77, 27–33. 10.1016/j.msec.2017.03.248 28532029

[B3] Akter N. Radiman S. Mohamed F. Reza M. I. (2013). Self-assembled potential bio nanocarriers for drug delivery. Mini Rev. Med. Chem. 13, 1327–1339. 10.2174/1389557511313090007 23544469

[B4] Alakhova D. Y. Kabanov A. V. (2014). Pluronics and MDR reversal: an update. Mol. Pharm. 11, 2566–2578. 10.1021/mp500298q 24950236 PMC4122590

[B5] Alfarouk K. O. Stock C. M. Taylor S. Walsh M. Muddathir A. K. Verduzco D.. (2015). Resistance to cancer chemotherapy: failure in drug response from ADME to P-gp. Cancer Cell Int. 15, 71. 10.1186/s12935-015-0221-1 26180516 PMC4502609

[B6] Arima K. Kakinuma A. Tamura G. (1968). Surfactin, a crystalline peptidelipid surfactant produced by *Bacillus subtilis*: isolation, characterization and its inhibition of fibrin clot formation. Biochem. Biophys. Res. Commun. 31, 488–494. 4968234 10.1016/0006-291x(68)90503-2

[B7] Banat I. M. Franzetti A. Gandolfi I. Bestetti G. Martinotti M. G. Fracchia L.. (2010). Microbial biosurfactants production, applications and future potential. Appl. Microbiol. Biotechnol. 87, 427–444. 10.1007/s00253-010-2589-0 20424836

[B8] Banik B. L. Fattahi P. Brown J. L. (2016). Polymeric nanoparticles: the future of nanomedicine. Nanomed. Nanobiotechnol. 8, 271–299. 10.1002/wnan.1364 26314803

[B9] Bei D. Meng J. Youan B. B. (2010). Engineering nanomedicines for improved melanoma therapy: progress and promises. Nanomedicine 5, 1385–1399. 10.2217/nnm.10.117 21128721 PMC3685319

[B10] Bernheimer A. W. Avigad L. S. (1970). Nature and properties of a cytolytic agent produced by *Bacillus subtilis*. J. Gen. Microbiol. 61, 361–369. 10.1099/00221287-61-3-361 4992273

[B11] Boettcher C. Kell H. Holzwarth J. F. Vater J. (2010). Flexible loops of thread-like micelles are formed upon interaction of L-alpha-dimyristoyl-phosphatidylcholine with the biosurfactant surfactin as revealed by cryo-electron tomography. Biophys. Chem. 149, 22–27. 10.1016/j.bpc.2010.03.006 20406718

[B12] Bouffioux O. Berquand A. Eeman M. Paquot M. Dufrene Y. F. Brasseur R.. (2007). Molecular organization of surfactin-phospholipid monolayers: effect of phospholipid chain length and polar head. Biochim. Biophys. Acta 1768, 1758–1768. 10.1016/j.bbamem.2007.04.015 17532292

[B13] Buchoux S. Lai-Kee-Him J. Garnier M. Tsan P. Besson F. Brisson A.. (2008). Surfactin-triggered small vesicle formation of negatively charged membranes: a novel membrane-lysis mechanism. Biophys. J. 95, 3840–3849. 10.1529/biophysj.107.128322 18515378 PMC2553101

[B14] Byeon S. E. Lee Y. G. Kim B. H. Shen T. Lee S. Y. Park H. J.. (2008). Surfactin blocks NO production in lipopolysaccharide-activated macrophages by inhibiting NF-kappaB activation. J. Microbiol. Biotechnol. 18, 1984–1989. 10.4014/jmb.0800.189 19131703

[B15] Cao X. H. Wang A. H. Wang C. L. Mao D. Z. Lu M. F. Cui Y. Q.. (2010). Surfactin induces apoptosis in human breast cancer MCF-7 cells through a ROS/JNK-mediated mitochondrial/caspase pathway. Chem. Biol. Interact. 183, 357–362. 10.1016/j.cbi.2009.11.027 19954742

[B16] Cao X. H. Zhao S. S. Liu D. Y. Wang Z. Niu L. L. Hou L. H.. (2011). ROS-Ca^2+^ is associated with mitochondria permeability transition pore involved in surfactin-induced MCF-7 cells apoptosis. Chem. Biol. Interact. 190, 16–27. 10.1016/j.cbi.2011.01.010 21241685

[B17] Cao X. Wang A. H. Jiao R. Z. Wang C. L. Mao D. Z. Yan L.. (2009). Surfactin induces apoptosis and G(2)/M arrest in human breast cancer MCF-7 cells through cell cycle factor regulation. Cell Biochem. Biophys. 55, 163–171. 10.1007/s12013-009-9065-4 19669740

[B18] Carrillo C. Teruel J. A. Aranda F. J. Ortiz A. (2003). Molecular mechanism of membrane permeabilization by the peptide antibiotic surfactin. Biochim. Biophys. Acta 1611, 91–97. 10.1016/S0005-2736(03)00029-4 12659949

[B19] Chen W. C. Juang R. S. Wei Y. H. (2015). Applications of a lipopeptide biosurfactant, surfactin, produced by microoganisms. Biochem. Eng. J. 103, 158–169. 10.1016/j.bej.2015.07.009

[B20] Chiewpattanakul P. Phonnok S. Durand A. Marie E. Thanomsub B. W. (2010). Bioproduction and anticancer activity of biosurfactant produced by the dematiaceous fungus *Exophiala dermatitidis* SK80. J. Microbiol. Biotechnol. 20, 1664–1671. 10.4014/jmb.1007.07052 21193821

[B21] Cho K. Wang X. Nie S. Chen Z. G. Shin D. M. (2008). Therapeutic nanoparticles for drug delivery in cancer. Clin. Cancer Res. 14, 1310–1316. 10.1158/1078-0432.CCR-07-1441 18316549

[B22] Conti E. Stachelhaus T. Marahiel M. A. Brick P. (1997). Structural basis for the activation of phenylalanine in the non-ribosomal biosynthesis of gramicidin S. EMBO J. 16, 4174–4183. 9250661 10.1093/emboj/16.14.4174 PMC1170043

[B23] Damasceno B. P. Dominici V. A. Urbano I. A. Silva J. A. Araújo I. B. Santos-Magalhães N. S.. (2012). Amphotericin B microemulsion reduces toxicity and maintains the efficacy as an antifungal product. J. Biomed. Nanotechnol. 8, 290–300. 10.1166/jbn.2012.1374 22515080

[B24] Das P. Mukherjee S. Sen R. (2008). Antimicrobial potential of a lipopeptide biosurfactant derived from a marine *Bacillus circulans*. J. Appl. Microbiol. 104, 1675–1684. 10.1111/j.1365-2672.2007.03701 18194244

[B25] Davis M. E. Chen Z. G. Shin D. M. (2008). Nanoparticle therapeutics: an emerging treatment modality for cancer. Nat. Rev. Drug Discov. 7, 771–782. 10.1038/nrd2614 18758474

[B26] Dehghan-Noude G. Housaindokht M. Bazzaz B. S. (2005). Isolation, characterization, and investigation of surface and hemolytic activities of a lipopeptide biosurfactant produced by *Bacillus subtilis* ATCC 6633. J. Microbiol. 43, 272–276. 15995646

[B27] Deleu M. Lorent J. Lins L. Brasseur R. Braun N. El Kirat K.. (2013). Effects of surfactin on membrane models displaying lipid phase separation. Biochim. Biophys. Acta 1828, 801–815. 10.1016/j.bbamem.2012.11.007 23159483

[B28] Demain A. L. Sanchez S. (2009). Microbial drug discovery: 80 years of progress. J. Antibiot. 62, 5–16. 10.1038/ja.2008.16 19132062 PMC7094699

[B29] Dey G. Bharti R. Sen R. Mandal M. (2015). Microbial amphiphiles: a class of promising new-generation anticancer agents. Drug Discov. Today 20, 136–146. 10.1016/j.drudis.2014.09.006 25241656

[B30] Dieckmann R. Lee Y. O. van Liempt H. von Dohren H. Kleinkauf H. (1995). Expression of an active adenylate-forming domain of peptide synthetases corresponding to acyl-CoA-synthetases. FEBS Lett. 357, 212–216. 7805893 10.1016/0014-5793(94)01342-x

[B31] D'Souza C. Nakano M. M. Corbell N. Zuber P. (1993). Amino-acylation site mutations in amino acid-activating domains of surfactin synthetase: effects on surfactin production and competence development in *Bacillus subtilis*. J. Bacteriol. 175, 3502–3510 8501054 10.1128/jb.175.11.3502-3510.1993 PMC204750

[B32] Duarte C. Gudina E. J. Lima C. F. Rodrigues L. R. (2014). Effects of biosurfactants on the viability and proliferation of human breast cancer cells. AMB Express 4, 40. 10.1186/s13568-014-0040-0 24949273 PMC4052778

[B33] Dy G. K. Adjei A. A. (2013). Understanding, recognizing, and managing toxicities of targeted anticancer therapies. CA Cancer J. Clin. 63, 249–279. 10.3322/caac.21184 23716430

[B34] Fracchia L. Banat J. J. Cavallo M. Ceresa C. (2015). Potential therapeutic applications of microbial surface-active compounds. Bioengineering 2, 144–162. 10.3934/bioeng.2015.3.144

[B35] Fuma S. Fujishima Y. Corbell N. D'Souza C. Nakano M. M. Zuber P.. (1993). Nucleotide sequence of 5' portion of srfA that contains the region required for competence establishment in *Bacillus subtilus*. Nucleic Acids Res. 21, 93–97. 8441623 10.1093/nar/21.1.93 PMC309069

[B36] Geiger T. Clarke S. (1987). Deamidation, isomerization, and racemization at asparaginyl and aspartyl residues in peptides. J. Biol. Chem. 262, 785–794. 3805008

[B37] Glaser T. Han I. Wu L. Zeng X. (2017). Targeted nanotechnology in glioblastoma multiforme. Front. Pharmacol. 8:166. 10.3389/fphar.2017.00166 28408882 PMC5374154

[B38] Grau A. Gomez Fernandez J. C. Peypoux F. Ortiz A. (1999). A study on the interactions of surfactin with phospholipid vesicles. Biochim. Biophys. Acta 1418, 307–319. 10320682 10.1016/s0005-2736(99)00039-5

[B39] Greish K. (2010). Enhanced Permeability and Retention (EPR) effect for anticancer nanomedicine drug targeting, in Cancer Nanotechnology: Methods and Protocols, eds Grobmyer S. R. Moudgil B. M. (Gainsville, FL: Humana Press), 25–39. 10.1007/978-1-60761-609-2_3 20217587

[B40] Gudiña E. J. Rangarajan V. Sen R. Rodrigues L. R. (2013). Potential therapeutic applications of biosurfactants. Trends Pharmacol. Sci. 34, 667–675. 10.1016/j.tips.2013.10.002 24182625

[B41] Gudiña E. J. Teixeira J. A. Rodrigues L. R. (2016). Biosurfactants produced by marine microorganisms with therapeutic applications. Mar. Drugs 14:E38. 10.3390/md14020038 26901207 PMC4771991

[B42] Hamoen L. W. Venema G. Kuipers O. P. (2003). Controlling competence in *Bacillus subtilis*: shared use of regulators. Microbiology 149(Pt 1), 9–17. 10.1099/mic.0.26003-0 12576575

[B43] Hazra C. Bari S. Kundu D. Chaudhari A. Mishra S. Chatterjee A. (2014a). Ultrasound-assisted/biosurfactant-templated size-tunable synthesis of nano-calcium sulfate with controllable crystal morphology. Ultrason. Sonochem. 21, 1117–1131. 10.1016/j.ultsonch.2013.12.020 24412181

[B44] Hazra C. Kundu D. Chatterjee A. Chaudhari A. Mishra S. (2014b). Poly (methyl methacrylate)(core)–biosurfactant (shell) nanoparticles: Size controlled sub-100nm synthesis, characterization, antibacterial activity, cytotoxicity and sustained drug release behavior. Colloids Surf. A. Physicochem. Eng. Aspects 449, 96–113. 10.1016/j.colsurfa.2014.02.051

[B45] Heerklotz H. Seelig J. (2001). Detergent-like action of the antibiotic peptide surfactin on lipid membranes. Biophys. J. 81, 1547–1554. 10.1016/S0006-3495(01)75808-0 11509367 PMC1301632

[B46] Heerklotz H. Seelig J. (2007). Leakage and lysis of lipid membranes induced by the lipopeptide surfactin. Eur. Biophys. J. 36, 305–314. 10.1007/s00249-006-0091-5 17051366

[B47] Hilvo M. Denkert C. Lehtinen L. Muller B. Brockmoller S. Seppanen-Laakso T.. (2011). Novel theranostic opportunities offered by characterization of altered membrane lipid metabolism in breast cancer progression. Cancer Res. 71, 3236–3245. 10.1158/0008-5472.CAN-10-3894 21415164

[B48] Hosono K. Suzuki H. (1983). Acylpeptides, the inhibitors of cyclic adenosine 3',5'-monophosphate phosphodiesterase. III. Inhibition of cyclic AMP phosphodiesterase. J. Antibiot. 36, 679–683. 6307959 10.7164/antibiotics.36.679

[B49] Hsieh F. C. Li M. C. Lin T. C. Kao S. S. (2004). Rapid detection and characterization of surfactin-producing *Bacillus subtilis* and closely related species based on PCR. Curr. Microbiol. 49, 186–191. 10.1007/s00284-004-4314-7 15386102

[B50] Hwang Y.-H. Kim M.-S. Song I.-B. Park B.-K. Lim J.-H. Park S.-C. (2009). Subacute (28 day) toxicity of Surfactin C, a lipopeptide produced by *Bacillus subtilis*, in rats. J. Health Sci. 55, 351–355. 10.1248/jhs.55.351

[B51] Israelachvili J. (1994). The science and applications of emulsions - an overview. Colloids Surf A. 91, 1–8. 10.1016/0927-7757(94)02743-9

[B52] Jagur-Grodzinski J. (2009). Polymers for targeted and/or sustained drug delivery. Polym. Adv. Technol. 20, 595–606. 10.1002/pat.1304

[B53] Joe M. M. Bradeeba K. Parthasarathi R. Sivakumar P. K. Chaulan P. S. Tipayno S. (2012). Development of surfactin based nanoemulsion formulation from selected cooking oils: evaluation for antimicrobial activity against food associated microorganisms. J. Taiwan Inst. Chem. Eng. 43, 172–180. 10.1016/j.jtice.2011.08.008

[B54] Kakinuma A. Hori M. Isono M. Tamura G. Arima K. (1969). Determination of amino acid sequence in surfactin, a crystalline peptidolipid surfactant produced by *Bacillus subtilis*. Agric. Biol. Chem. 33, 971–977. 10.1080/00021369.1969.10859408

[B55] Kameda Y. Kanatomo S. (1968). Abstracts papers, The 88th Annual Meeting of Pharmaceutical Society of Japan, in The 88th Annual Meeting of Pharmaceutical Society of Japan (Nagoya), 431.

[B56] Kameda Y. Oira S. Matsui K. Kanatomo S. Hase T. (1974). Anti-tumor activity of Bacillus natto 5. Isolation and characterization of surfactin in the culture medium of Bacillus natto KMD 2311. Chem. Pharmaceut. Bull. 22, 938–944. 10.1248/cpb.22.938 4473150

[B57] Kapse-Mistry S. Govender T. Srivastava R. Yergeri M. (2014). Nanodrug delivery in reversing multidrug resistance in cancer cells. Front. Pharmacol. 5:159. 10.3389/fphar.2014.00159 25071577 PMC4090910

[B58] Kell H. Holzwarth J. F. Boettcher C. Heenan R. K. Vater J. (2007). Physicochemical studies of the interaction of the lipoheptapeptide surfactin with lipid bilayers of L-alpha-dimyristoyl phosphatidylcholine. Biophys. Chem. 128, 114–124. 10.1016/j.bpc.2007.03.005 17383076

[B59] Khodabandehloo H. Zahednasab H. Ashrafi Hafez A. (2016). Nanocarriers usage for drug delivery in cancer therapy. Iran J. Cancer Prev. 9:e3966. 10.17795/ijcp-3966 27482328 PMC4951761

[B60] Kikuchi T. Hasumi K. (2002). Enhancement of plasminogen activation by surfactin C: augmentation of fibrinolysis *in vitro* and *in vivo*. Biochim. Biophys. Acta 1596, 234–245. 10.1016/S0167-4838(02)00221-2 12007605

[B61] Kim S. Y. Kim J. Y. Kim S. H. Bae H. J. Yi H. Yoon S. H.. (2007). Surfactin from *Bacillus subtilis* displays anti-proliferative effect via apoptosis induction, cell cycle arrest and survival signaling suppression. FEBS Lett. 581, 865–871. 10.1016/j.febslet.2007.01.059 17292358

[B62] Kim W. Gates K. S. (1997). Evidence for thiol-dependent production of oxygen radicals by 4-methyl-5-pyrazinyl-3H-1,2-dithiole-3-thione (oltipraz) and 3H-1,2-dithiole-3-thione: possible relevance to the anticarcinogenic properties of 1,2-dithiole-3-thiones. Chem. Res. Toxicol. 10, 296–301. 10.1021/tx9601667 9084909

[B63] Kinsinger R. F. Kearns D. B. Hale M. Fall R. (2005). Genetic requirements for potassium ion-dependent colony spreading in *Bacillus subtilis*. J. Bacteriol. 187, 8462–8469. 10.1128/JB.187.24.8462-8469.2005 16321950 PMC1317027

[B64] Kluge B. Vater J. Salnikow J. Eckart K. (1988). Studies on the biosynthesis of surfactin, a lipopeptide antibiotic from *Bacillus subtilis* ATCC 21332. FEBS Lett. 231, 107–110. 3129307 10.1016/0014-5793(88)80712-9

[B65] Kraas F. I. Helmetag V. Wittmann M. Strieker M. Marahiel M. A. (2010). Functional dissection of surfactin synthetase initiation module reveals insights into the mechanism of lipoinitiation. Chem. Biol. 17, 872–880. 10.1016/j.chembiol.2010.06.015 20797616

[B66] Krishnan N. Velramar B. Pandiyan R. Velu R. K. (2017). Anti-pseudomonal and anti-endotoxic effects of surfactin-stabilized biogenic silver nanocubes ameliorated wound repair in streptozotocin-induced diabetic mice. Artif. Cells Nanomed. Biotechnol. 14, 1–12. 10.1080/21691401.2017.1324461 28503994

[B67] Kundu D. Hazra C. Chatterjee A. Chaudhari A. Mishra S. Kharat A. (2016). Surfactin-functionalized poly (methyl methacrylate) as an eco-friendly nano-adsorbent: from size-controlled scalable fabrication to adsorptive removal of inorganic and organic pollutants. RSC Adv. 6, 80438–80454. 10.1039/C6RA10804K

[B68] Kural F. H. Gürsoy R. N. (2011). Formulation and characterization of surfactin-containing self-microemulsifying drug delivery systems (SF-SMEDDS). Hacettepe Univ. J. Faculty Pharmacy 30, 171–186. Available online at: https://pdfs.semanticscholar.org/6107/07aafde9f56c2a14d7ee9ad5b0ff5991682f.pdf

[B69] Lambalot R. H. Gehring A. M. Flugel R. S. Zuber P. LaCelle M. Marahiel M. A.. (1996). A new enzyme superfamily - the phosphopantetheinyl transferases. Chem. Biol. 3, 923–936. 8939709 10.1016/s1074-5521(96)90181-7

[B70] Lawrence M. J. (1994). Surfactant systems: microemulsions and vesicles as vehicles for drug delivery. Eur. J. Drug Metab. Pharmacokinet. 19, 257–269. 7867669 10.1007/BF03188929

[B71] Lee J. H. Nam S. H. Seo W. T. (2012). The production of surfactin during the fermentation of cheonggukjang by potential probiotic *Bacillus subtilis* CSY191 and the resultant growth suppression of MCF-7 human breast cancer cells. Food Chem. 131, 1347–1354. 10.1016/j.foodchem.2011.09.133

[B72] Linne U. Marahiel M. A. (2000). Control of directionality in nonribosomal peptide synthesis: role of the condensation domain in preventing misinitiation and timing of epimerization. Biochemistry 39, 10439–10447. 10.1021/bi000768w 10956034

[B73] Liu J. F. Mbadinga S. M. Yang S. Z. Gu J. D. Mu B. Z. (2015). Chemical structure, property and potential applications of biosurfactants produced by *Bacillus subtilis* in petroleum recovery and spill mitigation. Int. J. Mol. Sci. 16, 4814–4837. 10.3390/ijms16034814 25741767 PMC4394451

[B74] Liu J. Zou A. Mu B. (2010). Toluidine blue: aggregation properties and distribution behavior in surfactin micelle solution. Colloids Surf. B Biointerfaces 75, 496–500. 10.1016/j.colsurfb.2009.09.025 19854627

[B75] Liu X. Tao X. Zou A. Yang S. Zhang L. Mu B. (2010). Effect of the microbial lipopeptide on tumor cell lines: apoptosis induced by disturbing the fatty acid composition of cell membrane. Protein Cell 1, 584–594. 10.1007/s13238-010-0072-4 21204010 PMC4875320

[B76] Maget-Dana R. Ptak M. (1992). Interfacial properties of surfactin. J. Colloid Interface Sci. 153, 285–291. 10.1016/0021-9797(92)90319-H

[B77] Maget-Dana R. Ptak M. (1995). Interactions of surfactin with membrane models. Biophys. J. 68, 1937–1943. 10.1016/S0006-3495(95)80370-X 7612835 PMC1282096

[B78] Maity J. P. Lin T. J. Cheng H. P. Chen C. Y. Reddy A. S. Atla S. B.. (2011). Synthesis of brushite particles in reverse microemulsions of the biosurfactant surfactin. Int. J. Mol. Sci. 12, 3821–3830. 10.3390/ijms12063821 21747709 PMC3131593

[B79] Marchant R. Banat I. M. (2012a). Biosurfactants: a sustainable replacement for chemical surfactants? Biotechnol. Lett. 34, 1597–1605. 10.1007/s10529-012-0956-x 22618240

[B80] Marchant R. Banat I. M. (2012b). Microbial biosurfactants: challenges and opportunities for future exploitation. Trends Biotechnol. 30, 558–565. 10.1016/j.tibtech.2012.07.003 22901730

[B81] Meena K. R. Dhiman R. Sharma A. Kanwar S. S. (2016). Applications of lipopeptide(s) from a Bacillus sp: an overview. Res. J. Recent Sci. 5, 50–54. Available online at: http://www.isca.in/rjrs/archive/v5/i11/9.ISCA-RJRS-2016-091.pdf

[B82] Morachis J. M. Mahmoud E. A. Almutairi A. (2012). Physical and chemical strategies for therapeutic delivery by using polymeric nanoparticles. Pharmacol. Rev. 64, 505–519. 10.1124/pr.111.005363 22544864 PMC3400833

[B83] Mulligan C. N. (2005). Environmental applications for biosurfactants. Environ. Pollut. 133, 183–198. 10.1016/j.envpol.2004.06.009 15519450

[B84] Murugan C. Rayappan K. Thangam R. Bhanumathi R. Shanthi K. Vivek R.. (2016). Combinatorial nanocarrier based drug delivery approach for amalgamation of anti-tumor agents in bresat cancer cells: an improved nanomedicine strategies. Sci. Rep. 6:34053. 10.1038/srep34053 27725731 PMC5057072

[B85] Nakano M. M. Zuber P. (1989). Cloning and characterization of srfB, a regulatory gene involved in surfactin production and competence in *Bacillus subtilis*. J. Bacteriol. 171, 5347–5353. 2507521 10.1128/jb.171.10.5347-5353.1989 PMC210372

[B86] Nakano M. M. Zuber P. (1990). Molecular biology of antibiotic production in Bacillus. Crit. Rev. Biotechnol. 10, 223–240. 10.3109/07388559009038209 1702690

[B87] Nakano M. M. Corbell N. Besson J. Zuber P. (1992). Isolation and characterization of sfp: a gene that functions in the production of the lipopeptide biosurfactant, surfactin, in *Bacillus subtilis*. Mol. Gen. Genet. 232, 313–321. 1557038 10.1007/BF00280011

[B88] Nakano M. M. Magnuson R. Myers A. Curry J. Grossman A. D. Zuber P. (1991). srfA is an operon required for surfactin production, competence development, and efficient sporulation in *Bacillus subtilis*. J. Bacteriol. 173, 1770–1778. 1847909 10.1128/jb.173.5.1770-1778.1991 PMC207329

[B89] Nambam J. S. Philip J. (2012). Effects of interaction of ionic and nonionic surfactants on self-assembly of PEO-PPO-PEO triblock copolymer in aqueous solution. J. Phys. Chem. B 116, 1499–1507. 10.1021/jp208902a 22216946

[B90] Nicoli S. Eeman M. Deleu M. Bresciani E. Padula C. Santi P. (2010). Effect of lipopeptides and iontophoresis on aciclovir skin delivery. J. Pharm. Pharmacol. 62, 702–708. 10.1211/jpp.62.06.0006 20636857

[B91] Nozhat Z. Asadi A. Zahri S. (2012). Properties of Surfactin C-15 nanopeptide and its cytotoxic effect on human cervix cancer (HeLa) cell line. J. Nanomater. 2012, 1–5. 10.1155/2012/526580

[B92] Obtel M. Lyoussi B. Tachfouti N. Pelissier S. M. Nejjari C. (2015). Using surveillance data to understand cancer trends: an overview in Morocco. Arch. Public Health 73, 45. 10.1186/s13690-015-0094-8 26528393 PMC4629314

[B93] Onaizi S. A. Nasser M. S. Twaiq F. A. (2012). Micellization and interfacial behavior of a synthetic surfactant-biosurfactant mixture. Colloids Surf. A Physicochem. Eng. Aspects 415, 388–393. 10.1016/j.colsurfa.2012.09.014

[B94] Park S. Y. Kim J. H. Lee Y. J. Lee S. J. Kim Y. (2013). Surfactin suppresses TPA-induced breast cancer cell invasion through the inhibition of MMP-9 expression. Int. J. Oncol. 42, 287–296. 10.3892/ijo.2012.1695 23151889

[B95] Peypoux F. Bonmatin J. M. Wallach J. (1999). Recent trends in the biochemistry of surfactin. Appl. Microbiol. Biotechnol. 51, 553–563. 10390813 10.1007/s002530051432

[B96] Preetha A. Banerjee R. Huilgol N. (2005). Surface activity, lipid profiles and their implications in cervical cancer. J. Cancer Res. Ther. 1, 180–186. 10.4103/0973-1482.19600 17998650

[B97] Reddy A. S. Chen C. Y. Chen C. C. Jean J. S. Chen H. R. Tseng M. J.. (2010). Biological synthesis of gold and silver nanoparticles mediated by the bacteria *Bacillus subtilis*. J. Nanosci. Nanotechnol. 10, 6567–6574. 10.1166/jnn.2010.2519 21137763

[B98] Reddy A. S. Chen C. Y. Chen C. C. Jean J. S. Fan C. W. Chen H. R.. (2009a). Synthesis of gold nanoparticles via an environmentally benign route using a biosurfactant. J. Nanosci. Nanotechnol. 9, 6693–6699. 10.1166/jnn.2009.1347 19908586

[B99] Reddy A. S. Chen C.-Y. Baker S. C. Chen C.-C. Jean J.-S. Fan C.-W. (2009b). Synthesis of silver nanoparticles using surfactin: a biosurfactant as stabilizing agent. Mater. Lett. 63, 1227–1230. 10.1016/j.matlet.2009.02.028

[B100] Rodrigues L. R. (2015). Microbial surfactants: fundamentals and applicability in the formulation of nano-sized drug delivery vectors. J. Colloid Interface Sci. 449, 304–316. 10.1016/j.jcis.2015.01.022 25655712

[B101] Rosenberg E. Ron E. Z. (1999). High- and low-molecular-mass microbial surfactants. Appl. Microbiol. Biotechnol. 52, 154–162. 10499255 10.1007/s002530051502

[B102] Sachdev D. P. Cameotra S. S. (2013). Biosurfactants in agriculture. Appl. Microbiol. Biotechnol. 97, 1005–1016. 10.1007/s00253-012-4641-8 23280539 PMC3555348

[B103] Sak K. (2012). Chemotherapy and dietary phytochemical agents. Chemother. Res. Pract. 2012:282570. 10.1155/2012/282570 23320169 PMC3539428

[B104] Satpute S. K. Bhuyan S. S. Pardesi K. R. Mujumdar S. S. Dhakephalkar P. K. Shete A. M.. (2010). Molecular genetics of biosurfactant synthesis in microorganisms. Adv. Exp. Med. Biol. 672, 14–41. 10.1007/978-1-4419-5979-9_2 20545271

[B105] Schwarzer D. Mootz H. D. Linne U. Marahiel M. A. (2002). Regeneration of misprimed nonribosomal peptide synthetases by type II thioesterases. Proc. Natl. Acad. Sci. U.S.A. 99, 14083–14088. 10.1073/pnas.212382199 12384573 PMC137840

[B106] Sen R. (2010). Surfactin: biosynthesis, genetics and potential applications, in Biosurfactants, ed Sen R. (New York, NY: Springer), 316–323. 10.1007/978-1-4419-5979-9_24 20545293

[B107] Sercombe L. Veerati T. Moheimani F. Wu S. Y. Sood A. K. Hua S. (2015). Advances and challenges of liposome assisted drug delivery. Front. Pharmacol. 6:286. 10.3389/fphar.2015.00286 26648870 PMC4664963

[B108] Sergeev I. N. (2005). Calcium signaling in cancer and vitamin D. J. Steroid Biochem. Mol. Biol. 97, 145–151. 10.1016/j.jsbmb.2005.06.007 16081284

[B109] Seydlova G. Svobodova J. (2008). Review of surfactin chemical properties and the potential biomedical applications. Cent. Eur. J. Med. 3, 123–133. 10.2478/s11536-008-0002-5

[B110] Seydlova G. Cabala R. Svobodova J. (2011). Surfactin-novel solutions for global issues, in Biomedical Engineering, Trends, Research and Technologies, (Rijeka: InTech), 306–330.

[B111] Shaligram N. S. Singhal R. S. (2010). Surfactin - a review on biosynthesis, fermentation, purification and applications. Food Technol. Biotechnol. 48, 119 Available online at: http://www.ftb.com.hr/images/pdfarticles/2010/April-June/48-119.pdf

[B112] Shen H. H. Lin T. W. Thomas R. K. Taylor D. J. Penfold J. (2011). Surfactin structures at interfaces and in solution: the effect of pH and cations. J. Phys. Chem. B 115, 4427–4435. 10.1021/jp109360h 21446656

[B113] Shim G. Y. Kim S. H. Han S. E. Kim Y. B. Oh Y. K. (2009). Cationic surfactin liposomes for enhanced cellular delivery of siRNA. Asian J. Pharmaceut. Sci. 4, 207–214. Available online at: http://www.asianjps.com:8080/Jweb_ajps/fileup/PDF/AJPS2009-4(4)-1.pdf

[B114] Sineriz F. Hommel R. Kleber H. (2001). Production of Biosurfactants. Encyclopedia of Life Support Systems 5.

[B115] Singh B. R. Dwivedi S. Al-Khedhairy A. A. Musarrat J. (2011). Synthesis of stable cadmium sulfide nanoparticles using surfactin produced by Bacillus amyloliquifaciens strain KSU-109. Colloids Surf. B Biointerfaces 85, 207–213. 10.1016/j.colsurfb.2011.02.030 21435848

[B116] Singla R. K. Dubey H. D. Dubey A. K. (2014). Therapeutic spectrum of bacterial metabolites. Indo Global J. Pharmaceut. Sci. 2, 52–64. Available online at: http://www.iglobaljournal.com/wp-content/uploads/2014/08/3.-Singla-et-al.-IGJPS-2014.pdf

[B117] Sivapathasekaran C. Das P. Mukherjee S. Saravanakumar J. Mandal M. Sen R. (2010). Marine bacterium derived lipopeptides: characterization and cytotoxic activity against cancer cell lines. Int. J. Pept. Res. Ther. 16, 215–222. 10.1007/s10989-010-9212-1

[B118] Steller S. Sokoll A. Wilde C. Bernhard F. Franke P. Vater J. (2004). Initiation of surfactin biosynthesis and the role of the SrfD-thioesterase protein. Biochemistry 43, 11331–11343. 10.1021/bi0493416 15366943

[B119] Thimon L. Peypoux F. Maget-Dana R. Roux B. Michel G. (1992). Interactions of bioactive lipopeptides, iturin A and surfactin from *Bacillus subtilis*. Biotechnol. Appl. Biochem. 16, 144–151. 1457050

[B120] Tsuge K. Ohata Y. Shoda M. (2001). Gene yerP, involved in surfactin self-resistance in *Bacillus subtilis*. Antimicrob. Agents Chemother. 45, 3566–3573. 10.1128/AAC.45.12.3566-3573.2001 11709341 PMC90870

[B121] Vater J. Stein T. Vollenbroich D. Kruft V. Wittmann-Liebold B. Franke P.. (1997). The modular organization of multifunctional peptide synthetases. J. Protein Chem. 16, 557–564. 9246644 10.1023/a:1026386100259

[B122] Wang C. L. Liu C. Niu L. L. Wang L. R. Hou L. H. Cao X. H. (2013). Surfactin-induced apoptosis through ROS-ERS-Ca^2+^-ERK pathways in HepG2 cells. Cell Biochem. Biophys. 67, 1433–1439. 10.1007/s12013-013-9676-7 23733672 PMC3838591

[B123] Wang C. L. Ng T. B. Cao X. H. Jiang Y. Liu Z. K. Wen T. Y. (2009). CLP induces apoptosis in human leukemia K562 cells through Ca^2+^ regulating extracellular-related protein kinase ERK activation. Cancer Lett. 276, 221–227. 10.1016/j.canlet.2008.11.007 19100683

[B124] Wang C. L. Ng T. B. Yuan F. Liu Z. K. Liu F. (2007). Induction of apoptosis in human leukemia K562 cells by cyclic lipopeptide from *Bacillus subtilis* natto T-2. Peptides 28, 1344–1350. 10.1016/j.peptides.2007.06.014 17643554

[B125] Weber T. Baumgartner R. Renner C. Marahiel M. A. Holak T. A. (2000). Solution structure of PCP, a prototype for the peptidyl carrier domains of modular peptide synthetases. Structure 8, 407–418. 10.1016/S0969-2126(00)00120-9 10801488

[B126] Wei Y. H. Chu I. M. (1998). Enhancement of surfactin production in iron-enriched media by *Bacillus subtilis* ATCC 21332. Enzyme Microb. Technol. 22, 724–728. 10.1016/S0141-0229(98)00016-7

[B127] Wei Y. H. Wang L. F. Chang J. S. (2004). Optimizing iron supplement strategies for enhanced surfactin production with Bacillus subtilis. Biotechnol. Prog. 20, 979–983. 10.1021/bp030051a 15176908

[B128] Wei Y. H. Wang L. F. Changy J. S. Kung S. S. (2003). Identification of induced acidification in iron-enriched cultures of *Bacillus subtilis* during biosurfactant fermentation. J. Biosci. Bioeng. 96, 174–178. 16233504 10.1016/s1389-1723(03)90121-6

[B129] WHO (2017). Cancer: Key Facts [Online]. World Health Organization Available online at: http://www.who.int/mediacentre/factsheets/fs297/en/ (Accessed April 12, 2017).

[B130] Wrasidlo W. Mielgo A. Torres V. A. Barbero S. Stoletov K. Suyama T. L.. (2008). The marine lipopeptide somocystinamide A triggers apoptosis via caspase 8. Proc. Natl. Acad. Sci. U.S.A. 105, 2313–2318. 10.1073/pnas.0712198105 18268346 PMC2268133

[B131] Yeh M. S. Wei Y. H. Chang J. S. (2005). Enhanced production of surfactin from *Bacillus subtilis* by addition of solid carriers. Biotechnol. Prog. 21, 1329–1334. 10.1021/bp050040c 16080719

[B132] Yu B. Tai H. C. Xue W. Lee L. J. Lee R. J. (2010). Receptor-targeted nanocarriers for therapeutic delivery to cancer. Mol. Membr. Biol. 27, 286–298. 10.3109/09687688.2010.521200 21028937 PMC3789246

[B133] Yuan Y. Cai T. Xia X. Zhang R. Chiba P. Cai Y. (2016). Nanoparticle delivery of anticancer drugs overcomes multidrug resistance in breast cancer. Drug Deliv. 23, 3350–3357. 10.1080/10717544.2016.1178825 27098896

[B134] Zhang Y. Liu C. Dong B. Ma X. Hou L. Cao X.. (2015). Anti-inflammatory activity and mechanism of surfactin in lipopolysaccharide-activated macrophages. Inflammation 38, 756–764. 10.1007/s10753-014-9986-y 25331175

[B135] Zwicke G. L. Mansoori G. A. Jeffery C. J. (2012). Utilizing the folate receptor for active targeting of cancer nanotherapeutics. Nano Rev. 3:18496. 10.3402/nano.v3i0.18496 23240070 PMC3521101

